# Changes in the allocation of endogenous strigolactone improve plant biomass production on phosphate‐poor soils

**DOI:** 10.1111/nph.14847

**Published:** 2017-10-30

**Authors:** Guowei Liu, Johannes Pfeifer, Rita de Brito Francisco, Aurelia Emonet, Marina Stirnemann, Christian Gübeli, Olivier Hutter, Joëlle Sasse, Christian Mattheyer, Ernst Stelzer, Achim Walter, Enrico Martinoia, Lorenzo Borghi

**Affiliations:** ^1^ Department of Plant and Microbial Biology University of Zurich Zollikerstrasse 107 Zurich 8008 Switzerland; ^2^ Institute of Agricultural Sciences ETH Zurich Universitätstrasse 2 Zurich 8092 Switzerland; ^3^ Département de Biologie Moléculaire Végétale Faculté de Biologie et Médecine Biophore Lausanne CH‐1015 Switzerland; ^4^ Carnegie Institution for Science 1530 P Street NW Washington DC 20005 USA; ^5^ Goethe‐Universität Frankfurt am Main Theodor‐W.‐Adorno‐Platz 1 Frankfurt am Main 60323 Germany

**Keywords:** auxin, mycorrhization, petunia, phosphate uptake, plant biomass, PLEIOTROPIC DRUG RESISTANCE1 (PDR1), strigolactone, strigolactone transport

## Abstract

- Strigolactones (SLs) are carotenoid‐derived phytohormones shaping plant architecture and inducing the symbiosis with endomycorrhizal fungi. In *Petunia hybrida*, SL transport within the plant and towards the rhizosphere is driven by the ABCG‐class protein PDR1. *PDR1* expression is regulated by phytohormones and by the soil phosphate abundance, and thus SL transport integrates plant development with nutrient conditions.
- We overexpressed *PDR1* (PDR1 OE) to investigate whether increased endogenous SL transport is sufficient to improve plant nutrition and productivity. Phosphorus quantification and nondestructive X‐ray computed tomography were applied. Morphological and gene expression changes were quantified at cellular and whole tissue levels via time‐lapse microscopy and quantitative PCR.
- PDR1 OE significantly enhanced phosphate uptake and plant biomass production on phosphate‐poor soils. PDR1 OE plants showed increased lateral root formation, extended root hair elongation, faster mycorrhization and reduced leaf senescence. PDR1 overexpression allowed considerable SL biosynthesis by releasing SL biosynthetic genes from an SL‐dependent negative feedback.
- The increased endogenous SL transport/biosynthesis in PDR1 OE plants is a powerful tool to improve plant growth on phosphate‐poor soils. We propose PDR1 as an as yet unexplored trait to be investigated for crop production. The overexpression of PDR1 is a valuable strategy to investigate SL functions and transport routes.

## Introduction

Strigolactones (SLs) are recently characterized phytohormones that play a multitude of roles during plant development and plant–microbial interactions. Initially discovered as germination stimulants of parasitic weeds (Cook *et al*., 1966), nowadays it is known that SLs regulate plant shoot and root architectures (reviewed in Al‐Babili & Bouwmeester, 2015), leaf senescence (Yamada *et al*., 2014), responses to biotic and abiotic stresses (Ha *et al*., 2014; Torres‐Vera *et al*., 2014), cytoskeletal dynamics, auxin transport (Shinohara *et al*., 2013; Pandya‐Kumar *et al*., 2014) and hyphal branching of arbuscular mycorrhizal fungi (AMF) (Akiyama *et al*., 2005). SLs are carotenoid derivatives synthesized via a pathway starting in plastids with the all‐trans‐β‐carotene/9‐cis‐β‐carotene isomerase D27 (reviewed in Lopez‐Obando *et al*., 2015). Two dioxygenases, CAROTENOID CLEAVAGE DIOXYGENASE 7 (CCD7)/MORE AXILLARY GROWTH 3 (MAX3)/DECREASED APICAL DOMINANCE 3 (DAD3) and CCD8/MAX4/DAD1, then synthesize the first bioactive SL precursor, carlactone. As a further step, plant‐specific members of cytochrome P450 mono‐oxygenases, MORE AXILLARY BRANCHES1 (MAX1) and MAX1 homologs produce canonical SLs such as orobanchol and ent‐2′‐epi‐5‐deoxystrigol (Zhang *et al*., 2014a), respectively most abundant in *Petunia hybrida* and *Oryza sativa* (Kretzschmar *et al*., 2012; Xie *et al*., 2013), or carlactonoic acid derivatives in *Arabidopsis thaliana* (Abe *et al*., 2014; Seto *et al*., 2014). SL synthesis takes place in several plant tissues: root tips, stem nodes, and along the root and shoot vasculature (Lopez‐Obando *et al*., 2015). Despite the ubiquitous biosynthesis, grafting experiments and tracking of SLs and of the SL‐mimicking molecule GR24 showed that SLs (or their precursors) move from the root to the shoot (Domagalska & Leyser, 2011; Sasse *et al*., 2015; Xie *et al*., 2015). However, wild‐type scions grafted on mutant root stocks do not show SL‐related phenotypes, supporting the hypothesis that shoots can produce enough SLs to regulate their architecture. *PLEIOTROPIC DRUG RESISTANCE1* (*PDR1*) from *P. hybrida* is the main player for SL shootward transport and SL release to the soil. PDR1 is apically localized in the plasma membrane of cortex cells in root tips and outer‐laterally localized in hypodermal passage cells (HPCs), the entry point of mycorrhizal fungi (Sasse *et al*., 2015). The high activity of the *PDR1* promoter (*pPDR1*) at the base of lateral axils and the bushy shoots of *pdr1* ko mutants suggest that SL transport has an important role in inhibiting lateral bud outgrowth (Kretzschmar *et al*., 2012).

Biosynthesis and transport of SLs and consequently SL amounts and allocation *in planta* and in the rhizosphere are regulated by external and internal cues. An important external factor is the availability of inorganic phosphate (Pi) in the soil (reviewed in Brewer *et al*., 2013). On Pi‐poor substrates, plants react to starvation by inducing SL exudation into the soil as a beacon for AMF; by lateral root formation and root hair elongation for improving the rhizosphere exploration; and by inhibiting shoot lateral branching. It has been shown that *PDR1* and *DAD1* expression levels are both up‐regulated by low Pi conditions as well as by auxins (Lopez‐Raez *et al*., 2008; Kretzschmar *et al*., 2012), thus suggesting that SLs act as integrators of plant nutrient uptake with plant growth regulation.

In modern agriculture, fertilization with phosphorus (P) from mineral sources is required to achieve high crop yields (Tilman, 1999; Roy‐Bolduc & Hijri, 2012). The commonly fertilized P form is soluble Pi, which is readily available to the plant. Arable soils in Europe, parts of Asia and America often contain surplus amounts of P (Cordell *et al*., 2009). The current, massive input of soluble Pi is not sustainable, as a result of depletion of global P reservoirs and eutrophication of waters by runoff from agricultural lands (Scholz & Wellmer, 2013; Reijnders, 2014). In addition, crops can typically only utilize between 10% and 25% of the fertilized Pi (Cordell *et al*., 2009), because of its slow diffusion and adherence to soil particles. Plant Pi utilization efficiency can therefore be effectively improved, with simultaneous lowering of environmental risk, by an increase in root surface area realized via lateral roots, root hairs, cluster roots (Neumann & Martinoia, 2002) and mycorrhizal hyphae. SL‐focused strategies, if targeted to tailored crops, might also accelerate Pi uptake by promoting mycorrhiza and root hair growth and can therefore help to increase the availability of Pi for food production while simultaneously increasing the sustainability of crop production.

Here we report that the overexpression of PDR1 causes the reallocation of endogenous SL in both roots and shoots. This change improves plant biomass production on Pi‐poor soils compared with the wild‐type because of enhanced nutrient uptake caused by a larger lateral root system, postponed leaf senescence, higher density and increased length of root hairs, as well as faster mycorrhization. PDR1 overexpression therefore provides a chance to increase plant yield in phosphate‐scarce soils. The possible uses of plants with enhanced SL production and transport are discussed.

## Materials and Methods

### Plant growth and generation of PDR1 OE lines

The cloning of PDR1 OE, a GFP‐PDR1 protein fusion driven by the *35SCaMV* promoter, was previously described by Sasse *et al*. (2015). Three independent PDR1 OE lines have been used for this and previous research and confirmed for GFP‐PDR1 gene and protein expression. *P. hybrida* W115 (Mitchell) growth was tested in six specific soil mixtures, which differed in Pi content (see details in Table 1) and in the presence or absence of mycorrhizal fungi, in order to study the effects of increased lateral root density and mycorrhiza on plant biomass development and Pi uptake. *P. hybrida* W115 (wild‐type background of PDR1 OE), PDR1 OE, *pdr1* ko (Kretzschmar *et al*., 2012) and W115 × W138 (wild‐type background of *pdr1* ko) plants were grown under long‐day conditions (16 : 8 h, light : dark regime), at 60% relative humidity and 25°C on different soil mixtures. These were: natural soil (from University of Zurich botanical garden, with naturally occurring mycorrhizal fungi); clay granules (Oil Dri US‐Special, Chicago, IL, USA) and mineral soil (subsoil, also known as B‐soil horizon from University of Zurich botanical garden). Also soil mixes were used, as follows: natural soil mix (70% natural soil and 30% mineral soil) and Claymin (50% clay and 50% mineral soil). For clay+ and Claymin+, respective substrates were supplemented with half a teaspoon in 500 ml substrate of a sand inoculum of *Rizophagus irregularis*, a common AMF frequently used for inoculation studies (Martin *et al*., 2008). The inoculum was added 2 wk after plant germination with a low Pi inoculation medium prepared as previously described (Reddy *et al*., 2007), so that a total Pi amount of 0.112 mg was added to the initial 0.46 mg per pot (i.e. a total of 0.001 g l^−1^). The pot volume used for all experiments was 500 ml. Alternatively, *P. hybrida* seeds were plated on 0.85% (w/v) Phyto Agar (Duchefa, Haarlem, the Netherlands) medium containing 2.2 g l^−1^ Murashige and Skoog (half‐strength MS (§ MS)) medium (Duchefa) at 21°C. Low‐Pi MS medium for root experiments contained 0.25 mM instead of 1.25 mM KH_2_PO_4_ as in § MS. Clay was chosen against mineral soil and full soil for analyses on the root system architecture because of easier washing away from roots for quantification of mycorrhization while at the same time keeping soil humidity more constant than mineral soil or sand.

**Table 1:** Phosphate content and description of the different soils used in this study

Substrate name	Substrate description	Inorganic phosphate (Pi) content (mg l^−1^)
Full natural soil	100% soil with naturally occurring mycorrhiza	1.61 ± 0.17
Natural soil mix	70% natural soil plus 30% mineral soil	1.29
Clay	100% clay	0.92 ± 0.07
Clay+	Clay added with *Rhizophagus irregularis* inoculum	1.03
Claymin	50% clay, 50% mineral soil	0.78
Claymin+	Claymin added with *Rhizophagus irregularis* inoculum	0.89
Mineral soil	100% mineral soil	0.53 ± 0.05

Values of Pi are means ± SE.

## Results

### Overexpression of *PDR1* induces fast mycorrhization and increased plant biomass production, and up‐regulates SL biosynthesis

**Figure 1:**
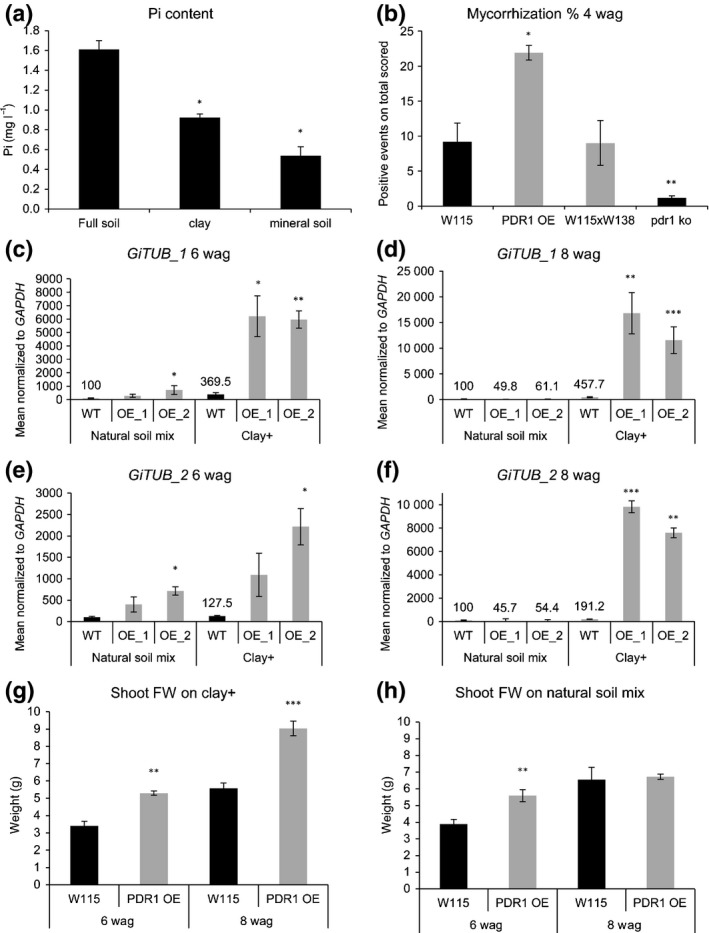
Biomasses and mycorrhization rates of PDR1 OE and wild‐type *Petunia hybrida* plants on soils containing different amounts of inorganic phosphate (Pi). (a) Pi abundances in soil, clay and mineral soil. (b) Mycorrhization rates at 4 wk after germination (wag) on natural soil mix in wild‐type (W115, WT), PDR1 OE (OE), the wild‐type background for *pdr1* ko (W115 × W138) and *pdr1* ko. (c, d) *Glomus intraradices* (now *Rhizophagus irregularis*) *TUBULIN_1 (GiTUB_1)* expression levels in wild‐type and PDR1 OE roots at 6 and 8 wag. (e, f) *Glomus intraradices* (now *Rhizophagus irregularis*) *TUBULIN_2 (GiTUB_2)* expression levels in wild‐type and PDR1 OE roots at 6 and 8 wag. (g) Shoot biomass production on clay+. (h) Shoot biomass production on natural soil mix. Values are means ± SE. *, *P *<* *0.05; **, *P *<* *0.005; ***, *P *<* *0.0005.

Mycorrhization is initiated by SL exudation which induces AMF hyphal branching towards the plant root (Akiyama *et al*., 2005). We therefore assessed whether PDR1 OE plants have higher mycorrhization levels than the wild‐type. As mycorrhization levels are influenced by soil Pi conditions (Breuillin *et al*., 2010), we quantified mycorrhization in different soils, from the Pi‐richer ‘natural soil mix’ to the Pi‐poorer ‘mineral soil’, going through ‘clay’ and mixed substrates with intermediate Pi amounts (see Fig. 1a, Table 1 and Supporting Information Methods S1; Table S1 for statistical analysis).

To test if *PDR1* expression levels influence mycorrhization, we first quantified it via the grid method on natural soil mix (see the Materials and Methods section). PDR1 OE roots exhibited a mycorrhization rate of 20% compared with 10% for wild‐type plants; *pdr1* ko plants scored < 2% (Fig. 1b). As the grid method does not easily allow the quantification of mycorrhization along the whole root, we further scored mycorrhizal rates via qPCR with specific mycorrhizal markers: *GiTUB_1* (primers kindly provided by Prof. Didier Reinhardt) and *GiTUB_2* (Alkan *et al*., 2004). On natural soil mix, PDR1 OE plants conserved stronger than the wild‐type *GiTUB* expression levels until 6 wk after germination (wag) and only on clay+ (100% clay supplied with the AMF *Rhizophagus irregularis*) up to 8 wag (Fig. 1c–f). In detail, relative to the wild‐type on natural soil mix, on clay+ wild‐type plants showed four‐ to fivefold higher *GiTUB_1* expression while PDR1 OE plants showed 60‐ to 150‐fold higher expression at 6 and 8 wag, respectively (Fig. 1c,d). With *GiTUB_2* the trend was similar on clay+: wild‐type plants scored 1.3‐ to twofold induction and PDR1 OE plants 22–100 times higher expression levels (Fig. 1e,f), confirming the higher mycorrhization capability of PDR1 OE on low‐Pi soils such as clay+. The different expression levels of *GiTUB1_1* and *GiTUB_2* are probably a result of the primer specificity. A BLAST analysis with *GiTUB_1* hit four different types of the genus *Glomus* (*G. clarum*,* G. claroideum*,* G. intraradices*,* G. diaphanum*) while *GiTUB_2* are specific for *G. intraradices* (now *Rhizophagus irregularis*). With both *GiTUB* primers, PDR1 OE scored six to 10 times stronger expression on natural soil mix at 6 wag, while at 8 wag the wild‐type scored higher mycorrhization than PDR1 OE, although with low statistical significance (Fig. 1d,f). Via the gridline quantification, we observed partly overlapping but underestimated trends, probably owing to the limited amount of root we could visualize on the total and to mycorrhization spatial heterogeneity (Gamper *et al*., 2008), both along the root and through the cortex layers of *P. hybrida* roots. In summary, we observed significantly higher mycorrhization only in PDR1 OE plants compared with the wild‐type at 6 wag on clay+ (Fig. S1a,b). The mycorrhizal structures observed via the grid method at 8 wag comprised mostly arbuscules (Fig. S1c–j) both on natural soil mix (88.6 ± 4.9%) and on clay+ (74.6 ± 8.3%). Of the nonarbuscular structures, no fungal vesicles were present on natural soil mix and 8.06 ± 0.92% (*n *=* *3) of vesicles were present on clay+.

**Figure 2:**
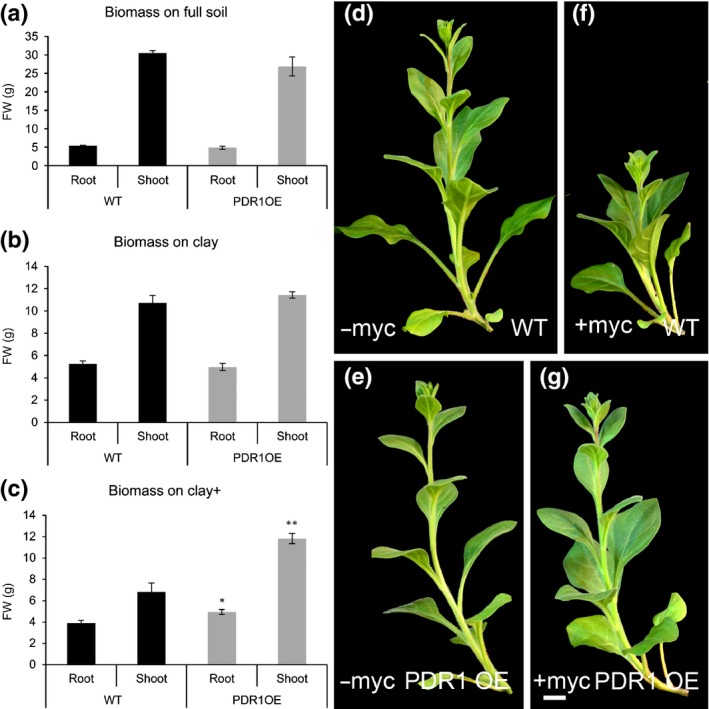
Correlation between genotypes, soils and Pi uptake in *Petunia hybrida* W115 and PDR1 OE plants. (a–c) FW (root and shoot) on full soil, clay and clay+ 8 wk after germination (wag). (d–g) Representative shoots of W115 and PDR1 OE plants grown on clay (−myc) and clay+ (+myc). Bars, 2 cm. Values are means ± SE. *, *P *<* *0.05; **, *P *<* *0.005.

Concomitantly with the long‐lasting, high mycorrhization rate of PDR1 OE plants on clay+ and the transient effect on natural soil mix, on clay+ we observed a significantly faster and long‐lasting increase of shoot biomass compared with the wild‐type (Fig. 1g) and a similar and transient increase on natural soil mix at 6 wag (Fig. 1h). As PDR1 OE shoot biomass on natural soil mix is lower than on clay+, the *R. irregularis* inoculum seems to induce a faster and more efficient mycorrhization than the naturally occurring mycorrhizal mix. To test if low Pi and AMF are both necessary for the growth advantage of PDR1 OE plants, we compared biomass production on full soil (our Pi richest soil) and on clay (without AMF). On full soil and on clay, no significant differences in biomass production could be observed 8 wag (Fig. 2a,b), while on clay+, used as positive control in this experimental setup, both shoot and root biomass production of PDR1 OE was again significantly higher than in the wild‐type (Fig. 2c). Interestingly, by comparing clay and clay+ results, it turned out that PDR1 OE can cope better with mycorrhization compared with the wild‐type, which is exploited to an extent by AMF in low‐Pi conditions (Fig. 2d–g). These results indicate that the faster mycorrhization on clay+ obtained through PDR1 OE is a major trait providing consistent advantage for plant growth, and that PDR1 OE plants can sustain more advantageous tradeoffs with AMF compared with the wild‐type. In order to assess whether the growth advantage on clay+ might be conferred by an enhanced plant Pi status, we determined the expression levels in roots of *P. hybrida PHOSPHATE TRANSPORTER 3 (PhPT3)* and *P. hybrida PHOSPHATE TRANSPORTER 5 (PhPT5)*, which are known to be strongly up‐regulated by mycorrhization (Breuillin *et al*., 2010). At 6 wag, the phosphate transporters were up‐regulated in PDR1 OE roots on both natural soil mix and clay+. At 8 wag, *PhPT3* and *PhPT5* were strongly induced under clay+ conditions and slightly down‐regulated in natural soil mix, according to the results obtained with the mycorrhization quantification via qPCR (Fig. S1k–n).

**Figure 3:**
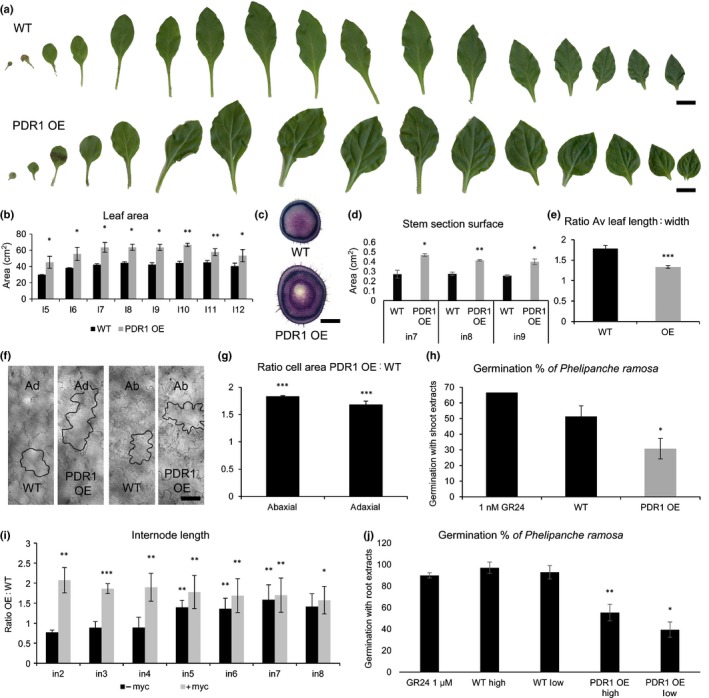
Shoot morphology and strigolactone (SL) quantification in wild‐type (W115 (WT)) and PDR1 OE *Petunia hybrida* plants grown on full soil. (a) Leaf series (from cotyledon to leaf number 15) in 2‐month‐old WT (W115) and PDR1 OE plants. (b) Leaf areas in W115 and PDR1 OE from leaf 5 (l5) to leaf 12 (l12). (c) Representative stem sections (node 9). (d) Significant differences in stem section areas in W115 to PDR1 OE from internode 7 (in7) to internode 9 (in9). (e) Ratio of leaf length : width in WT and PDR1 OE plants. (f) Representative light microscopy pictures of adaxial (ad) and abaxial (ab) epidermal cells in WT and PDR1 OE middle blades. (g) Ratio of PDR1 OE : WT epidermal cell areas. (h) Germination rates of *Phelipanche ramosa* induced by shoot extracts of WT and PDR1 OE plants. 1 nM GR24 as positive control. (i) PDR1 OE : WT ratio for internode (in) elongation ± mycorrhization. (j) Germination rates of *P. ramosa* induced by root extracts of WT and PDR1 OE plants. 1 μM GR24 as positive control. Root extracts diluted 10^−3^ (high) and 10^−5^ (low). Bars: (a) leaves, 1 cm; (c) stems, 3 mm; (f) epidermal cells, 20 μm. Values are means ± SE. *, *P *<* *0.05; **, *P *<* *0.005; ***, *P *<* *0.0005.

On clay+, neither wild‐type nor PDR1 OE plants showed lateral shoot growth during the first 8 wag (Fig. 2d–g), probably because of the limiting nutrient conditions, and therefore the higher biomass production of PDR1 OE plants is given by main stem and leaves. Interestingly, the main stem and leaves also account for the equal shoot biomass production on full soil (Fig. 2a), where wild‐type plants exhibit a stronger branching compared with PDR1 OE, as previously published (Sasse *et al*., 2015). We then analyzed in detail the shoot morphology of PDR1 OE on full soil, and we scored larger and rounder foliage surface and thicker stems in PDR1 OE than in the wild‐type (Fig. 3a–d). The leaf length : width ratio in PDR1 OE leaves was smaller than in the wild‐type (Fig. 3e). Epidermal cells in PDR1 OE leaves (middle blade) were larger than in the wild‐type (Fig. 3f,g).

To test if altered SL allocations in PDR1 OE shoots are responsible for the observed phenotypes, we assayed SL‐inducible germination of the parasitic weed *Phelipanche ramosa* using *P. hybrida* shoot extracts from 1‐month‐old PDR1 OE plantlets, where leaves represent 87% (± 3.5%; *n *=* *5) of the shoot biomass and no lateral buds are formed yet. The germination of *Phelipanche ramosa* seeds is very sensitive to SLs (up to four orders of magnitude higher than mass spectrometer detection limit; see Guillotin *et al*., 2017). However, it can be induced not only by SLs but also by isothiocyanates (Auger *et al*., 2012), present almost exclusively in Brassicaceae (Halkier & Gershenzon, 2006). Still, we tested for the presence of possible inhibitors or activators in our plant extracts. The germination ability of GR24 was assayed ± wild‐type, *pdr1 ko* and PDR1 OE extracts. In none of the analyzed cases could the negative effect of unknown molecules in the extracts from *P. hybrida* tissues override the results we scored with pure extracts (Fig. S2a,b). This bioassay operated with PDR1 OE plantlets showed that PDR1 OE leaves contain lower concentrations of SL than the wild‐type (Fig. 3h). By contrast, increased PDR1 OE stem thickness was compatible with SL‐induced cell division in the procambium, as reported in *A. thaliana* after exogenous GR24 treatments (Agusti *et al*., 2011). PDR1 OE stems also had longer internodes than those of the wild‐type; with or without mycorrhizal fungi in the soil (Fig. 3i) PDR1 OE internodes were 1.5‐ to two‐fold longer than in the wild‐type. As SL was reported to increase internode elongation in *Pisum sativum* (de Saint Germain *et al*., 2013), we propose that this phenotype in *P. hybrida* is a result of increased SL transport within/towards the procambium.

**Figure 4:**
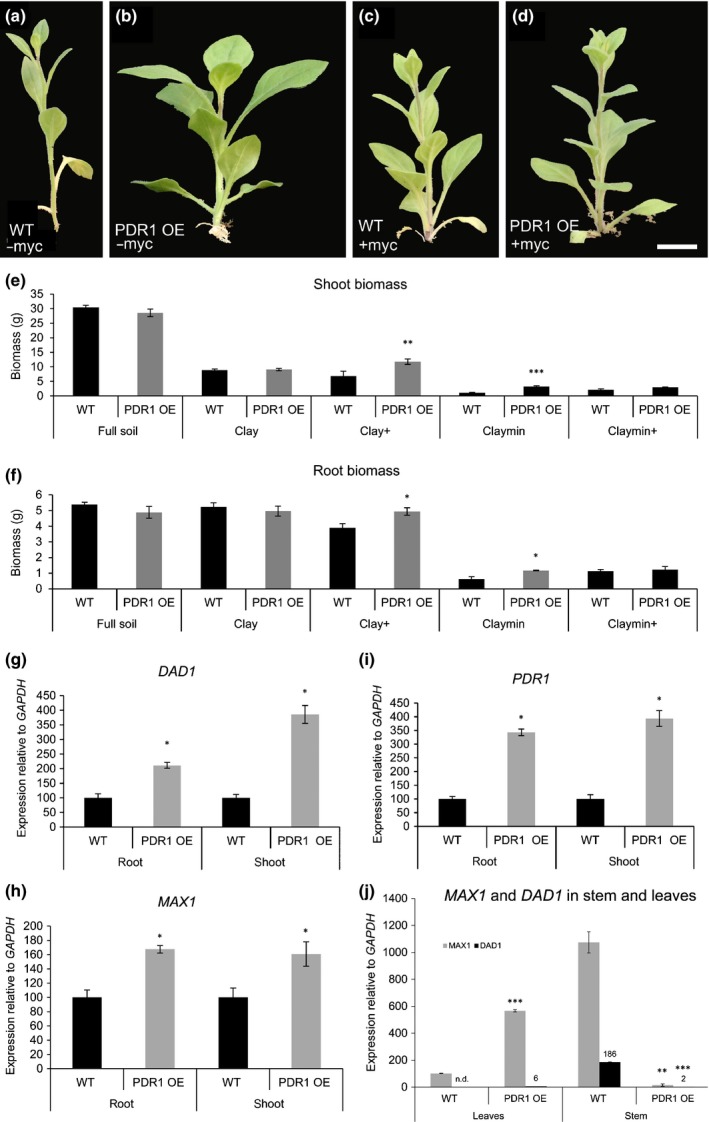
Biomass and gene expression levels in roots and shoots of wild‐type (W115 (WT)) and PDR1 OE *Petunia hybrida* plants. (a–d) Shoots of W115 and PDR1 OE on Claymin +/– mycorrhizal fungi. (e, f) Shoot and root biomasses in WT and PDR1 OE plants. (g, h) *DAD1* and *MAX1* expression levels in 6‐wk‐old PDR1 OE roots and shoots. (i) *PDR1* expression levels in 6‐wk‐old PDR1 OE plants. (j) *MAX1* and *DAD1* in stems and leaves of WT and PDR1 OE plants. Bars, 2 cm. Values are means ± SE. *, *P *<* *0.05; **, *P *<* *0.005; ***, *P *<* *0.0005.

The symbiosis between plants and mycorrhizal fungi is a continuous tradeoff as long as both organisms are beneficial to each other (Ryan *et al*., 2012). Therefore we tested PDR1 OE and wild‐type plants grown on soils with lower Pi concentrations than clay, where high levels of mycorrhization could lead to carbon exploitation from the host plant rather than to beneficial Pi upload from the fungus. On mineral soil (see Table 1), *P. hybrida* plants could not grow; therefore, we assayed plant biomass production at 8 wag on a substrate mixture called Claymin (50% clay plus 50% mineral soil ± AMF) (see the Materials and Methods section and Table 1). Under these conditions and in the absence of AMF, shoot and root growth of PDR1 OE plants were significantly higher than that of wild‐type plants (Fig. 4a,b,e,f). However, the addition of AMF did not cause significant increases in biomass production (Fig. 4c–f). In Claymin+ growth conditions, the costs of energy supply to the mycorrhiza seem to exceed the benefit obtained from the fungus. In none of the tested substrates could PDR1 OE plants reach the biomass production in full soil: still, on clay+ and Claymin, PDR1 OE plants could produce more shoot and root biomass than the wild‐type up to 8 wag (Fig. 4e,f). Consistently, an increase of Pi uptake is significant only in PDR1 OE plants grown on clay+ or Claymin (Fig. S2c).

The ability of PDR1 OE plants to obtain faster mycorrhization levels and longer/thicker stems suggests that SL biosynthesis might be induced to support the stronger SL transport/exudation driven by PDR1. We analyzed the expression levels of two SL biosynthetic genes, *DAD1* and *MAX1*, in three different PDR1 OE lines (Fig. S2d–f). Compared with the wild‐type, *PDR1*,* DAD1*, and *MAX1* are significantly up‐regulated (Fig. 4g–i) in shoots and roots of PDR1 OE plants. This result indicates that the overexpression of the transporter induces the SL biosynthesis pathway; SL biosynthesis is feedback‐inhibited by SL; and enhanced SL export from the site of its synthesis releases this feedback inhibition. To test if SL concentrations are changed in PDR1 OE roots, we assayed SL‐inducible germination of the parasitic weed *Phelipanche ramosa* using *P. hybrida* root extracts. PDR1 OE root extracts cannot induce *P. ramosa* germination as strongly as the wild‐type (Fig. 3j), showing that PDR1 OE roots are partially depleted in SL as a result of increased transport to the shoot and/or exudation to the soil. The *DAD1* and *MAX1* expression results in roots imply that their gene expression can be used as inversely proportional readouts of SL accumulation.

### A new SL export route from the leaves

To further investigate how PDR1 OE shoot phenotypes are related to SL reallocation, we assayed *DAD1* and *MAX1* expression to obtain an indirect and distinct quantification of SL in wild‐type and PDR1 OE 2‐month‐old (adult) plants. *DAD1* and *MAX1* were up‐regulated (Fig. 4j) in leaves of PDR1 OE plants, confirming the results obtained in 1‐month‐old PDR1 OE plantlet shoots. By contrast, *MAX1* and *DAD1* were strongly down‐regulated in PDR1 OE stems (Fig. 4j). This result suggests that SL accumulation in the stem (probably close to the nodes, as inferred from the SL‐related bud phenotype of PDR1 OE plants) might be a result not only of SL reallocation in the stem but also of SL depletion out of the leaves driven by PDR1. To test for the presence of this possible SL transport route from the leaf to the stem, we quantified SL from stem and leaf extracts with the *P. ramosa* germination assay in wild‐type, PDR1 OE and *pdr1* ko leaves.

**Figure 5:**
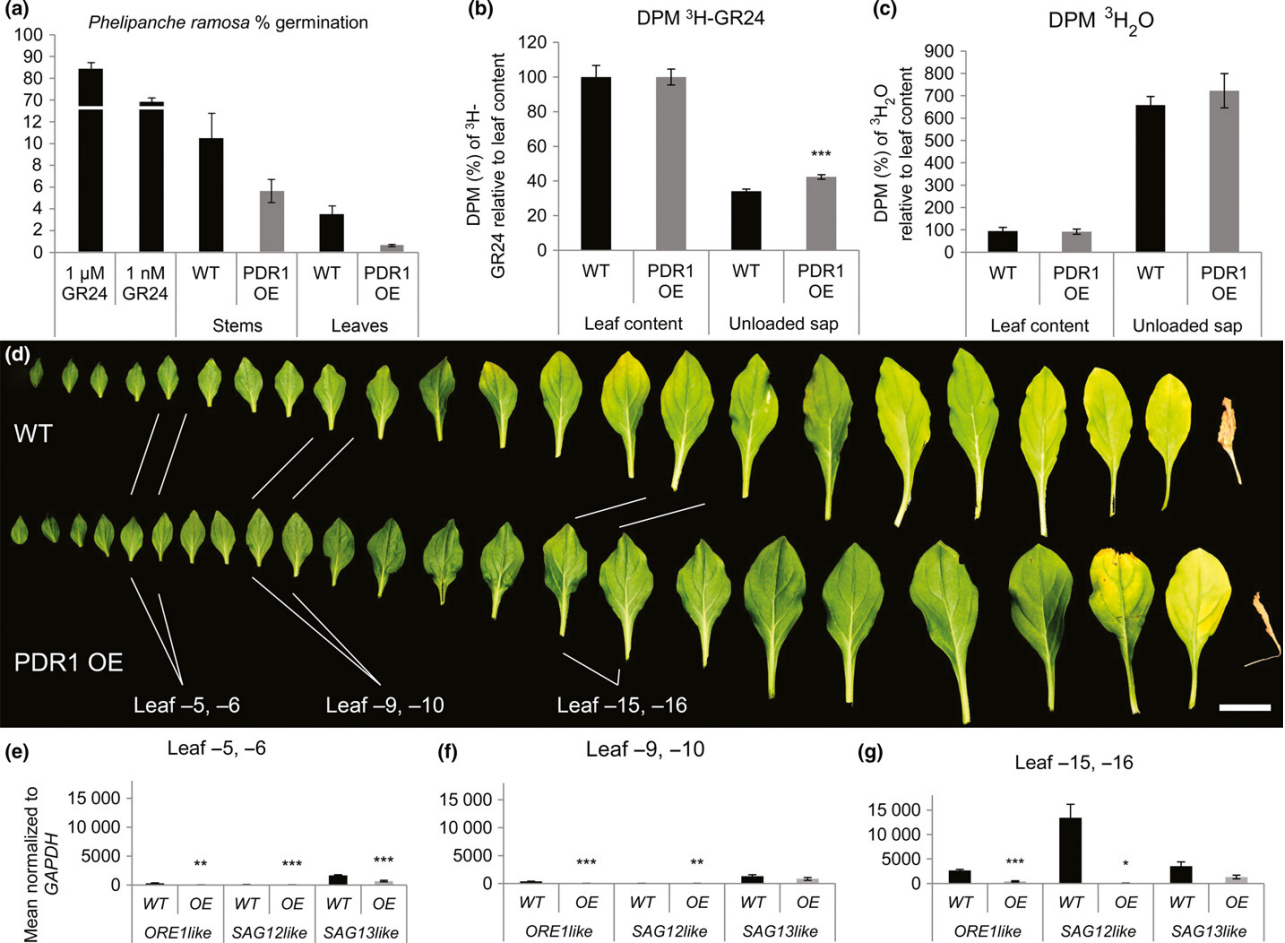
Semiquantitative strigolactone (SL) quantification, ^3^H‐GR24 transport and leaf senescence in wild‐type (WR) and PDR1 OE *Petunia hybrida* leaves. (a) Germination rate of *Phelipanche ramosa* seeds with leaf and stem extracts of WT and PDR1 OE plants. (b, c) Decays min^–1^ (DPM) of ^3^H‐GR24 present in leaf unloaded sap relative to ^3^H‐GR24 leaf content (b) and ^3^H_2_O leaf content (c). (d) Senescence‐related leaf phenotypes in 3‐month‐old WT and PDR1 OE plants from the last leaf grown before the transition to flowering time (leaf −1) up to leaf −23. (e–g) Gene expression levels of petunia *ORE1like*,*SAG12like* and *SAG13like* in leaves −5/−6, −9/−10 and −15/−16. Bars, 2 cm. Values are means ± SE. *, *P *<* *0.05; **, *P *<* *0.005; ***, *P *<* *0.0005.

Tissue extracts from 2‐month‐old PDR1 OE leaves and stems did not cause any germination of parasitic weeds in three attempts, probably because of low SL concentrations in adult shoots. However, two attempts with one order of magnitude lower dilutions of leaf extracts (see Methods S1) provided germination rates of between 1% and 4% (Fig. 5a). These results confirmed that SL concentrations in PDR1 OE shoots are lower than in the wild‐type, surprisingly also in stems where bud outgrowth is inhibited (Sasse *et al*., 2015). By contrast, the same experimental setup with extracts from *pdr1* ko leaves showed a higher germination rate than in the wild‐type (Fig. S3a), suggesting that PDR1 is necessary for transporting SLs out of the leaf.

We then compared SL transport in wild‐type, PDR1 OE and *pdr1* ko leaves by quantifying leaf loading and unloading of a radiolabeled SL‐mimicking molecule (^3^H‐GR24) and we compared leaf senescence, a known SL‐related phenotype (Figs 5b, S3b). Leaves were first incubated for 12 h in 1/2 MS + ^3^H‐GR24 and then transferred in cold 1/2 MS for an additional 10 h. ^3^H‐GR24 concentrations were scored after leaf loading and unloading. Wild‐type leaves released 26% (± 1.2%) of the loaded ^3^H‐GR24, while *pdr1* ko only released 18% (± 1.3%); PDR1 OE leaves released 46% (± 7.9%), while their wild‐type counterpart only released 32% (± 0.2%). No differences or opposite trends were scored in control transport experiments with tritiated water (Figs 5c, S3c). These results indicate that PDR1, and not transpiration or phloem flows, regulates SL transport out of the leaves. To test if GR24 and not its metabolites are exported from the leaf via PDR1, we directly quantified GR24 leaf loading and unloading via LC‐MS‐MS analyses (see Methods S1; Fig. S4a,b). The results showed GR24 stability in this time span, as also shown by Akiyama *et al*. (2010) and that the conditions of this experiment produced no detectable (below our detection limits) GR24 degradation products such as the ABC moiety or the hydrolyzed D‐ring (Fig. S4c,d), thus confirming the positive role of PDR1 in GR24 leaf export. Last but not least, we quantified senescence in PDR1 OE, *pdr1* ko and wild‐type leaves by visual examination, gene expression analysis and LC‐MS of nonfluorescent Chl catabolites (NCCs) (Berghold *et al*., 2004; Christ *et al*., 2016). Three months after germination, *P. hybrida* leaves were collected and compared. Wild‐type leaves showed senescence from the 11^th^ top leaf down (leaf −11), while in PDR1 OE, leaf senescence started being visible in marginal spots of the leaf blade in the 15^th^ leaf from the top (leaf −15) (Fig. 5d). Leaves of *pdr1* ko plants were significantly smaller than the relative wild‐type background (Fig. S3d,e) and started senescing between leaf −11 and leaf −14, at which point the wild‐type showed no senescing leaves. The gene expression of *P. hybrida ORE1‐like*,* SENESCENCE‐ASSOCIATED GENE 12‐like (SAG12like)* and *SAG13‐like* indicators of leaf senescence (Lohman *et al*., 1994; Breeze *et al*., 2011) confirmed that senescence emerges earlier in the wild‐type than in PDR1 OE plants, in leaves not showing wilting phenotypes (Fig. 5e,f). In the older leaves −15/−16 (Fig. 5g) the three genes were strongly up‐regulated in the wild‐type but significantly different only for *ORE1like* and *SAG12like*. Leaves −11 and −14 from *pdr1* ko plants showed an inverted behavior of the SL‐biosynthetic‐ and senescence‐related genes that we found deregulated in PDR1 OE leaves (Fig. S3f,g). MS analyses conducted on the same tissues confirmed the accumulation of NCC 806 and NCC 892 in wild‐type but not in PDR1 OE leaves (Fig. S5a–d). These results show that PDR1 OE leaves can export to the stem more SL than can the wild‐type, thus releasing *MAX1* and *DAD1* expression from the SL negative feedback and strongly postponing leaf senescence. Also, with the opposite results from the parallel analyses on *pdr1* ko plants, we propose that PDR1 regulates the transport of SL out of the leaves, either to the lateral buds or to the main stem.

### *PDR1* overexpression increases the root biomass

**Figure 6:**
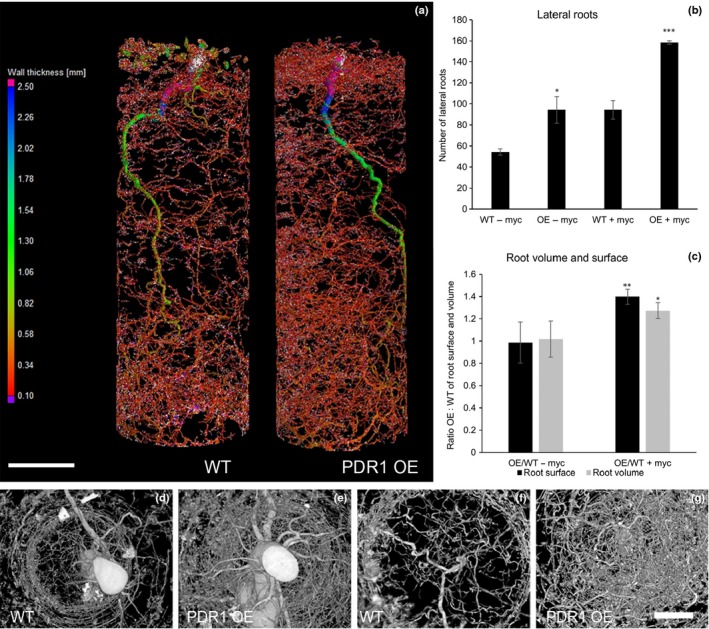
X‐ray computed tomography on wild‐type (WT) and PDR1 OE *Petunia hybrida* roots. X‐ray computed tomography on roots of 8‐wk‐old petunia plants. (a) Heat map (root thickness) of WT (left) and PDR1 OE (right) roots grown on clay+. (b) Quantification of lateral roots in WT and PDR1 OE roots ± myc. (c) Ratio of PDR1 OE : WT root volumes and root surfaces +/– myc. (d–g) Digital rendering (via Fiji software) of WT (d, f) and PDR1 OE (e, g) roots: (d, e) top view; (f, g) bottom view. Bars: (a) 4 cm; (d–g) 1 cm. Values are means ± SE. *, *P *<* *0.05.

**Figure 7:**
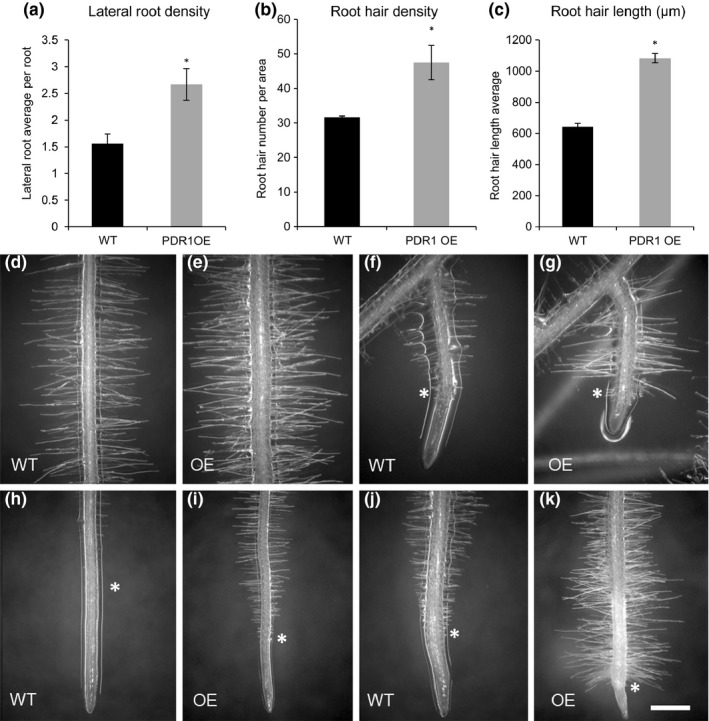
Root hair phenotypes in wild‐type (W115 (WT)) and PDR1 OE *Petunia hybrida* plants. Root hairs of 3‐wk‐old WT and PDR1 OE petunia plantlets grown on half‐strength MS agar plates. (a–c) Quantification of lateral root density, root hair density (area evaluated = 1.6 cm^2^) and root hair length in WT and PDR1 OE roots. (d, h) WT root segment: (d) differentiated (2 cm from the root tip); (h) above the root tip. (e, i) PDR1 OE root segment: (e) differentiated (2 cm from the root tip); (i) above the root tip. (f, j) WT lateral roots: (f) emerging; (j) elongated. (g, k) PDR1 OE lateral roots: (g) emerging; (k) elongated. In panels (a–k), the asterisk indicates the first root hair from the root tip. Bars, 400 μm. Values in (a–c) are means ± SE. *, *P *<* *0.05.

Strigolactones have been described to have an impact on lateral root and root hair formation (Kapulnik *et al*., 2011; Mayzlish‐Gati *et al*., 2012), factors that influence plant nutrition. We investigated whether PDR1 OE plants can produce a higher biomass than the wild‐type not only because of faster mycorrhization but also because of altered root structures that are possibly more efficient in nutrient uptake. Indeed, on Claymin in the absence of mycorrhiza, PDR1OE exhibit a higher biomass compared with the wild type (Fig. 4a–f). Using X‐ray computed tomography (see Methods S1 and Table S1) we screened root volumes, surfaces and the amounts of lateral roots on clay+, where we observed a significant increase of PDR1 OE biomass. At 6 wag the tomography did not reveal significant differences between wild‐type and PDR1 OE plants (Fig. S6a–c; Movies S1, S2). By contrast, at 8 wag, significantly more lateral roots could be observed in PDR1 OE plants (Fig. 6a). Interestingly, this difference was also visible in the absence of AMF (Fig. 6b,c; Movies S3, S4), while a significant increase in total root volume and surface was only measured when plants were grown with AMF, suggesting that without the input of AMF, lateral root growth was initiated but did not proceed as quickly. On clay+, a dense disc of lateral roots was present close to the soil surface in PDR1 OE plants, at a depth of 2–4 cm, but not in the wild‐type (Fig. 6d,e). Shallow roots are known to play an important role in nutrient uptake, as in several soils this is the most nutrient‐rich region (Liao *et al*., 2001; Lynch, 2013). Therefore, we suggest that PDR1 OE, in the presence of AMF, might also have an advantage by better scavenging surface‐close nutrients. These results show that the induction of lateral roots is linked to the mis‐regulation of *PDR1* expression and the consequent SL redistribution and not only to a higher rate of mycorrhization. Interestingly, 3‐wk‐old PDR1 OE seedlings grown under sterile conditions also have a higher number of lateral roots than wild‐type seedlings (Fig. 7a). This result confirms that *PDR1* overexpression induces lateral roots independently of the growth substrate and of symbioses with soil microbes.

### *PDR1* overexpression induces root hair elongation independently of the soil nutrient conditions

Root hairs contribute to nutrient uptake, especially of phosphate, nitrogen and water (Olah *et al*., 2005). Exogenous applications of GR24 were shown to induce root hair development in Arabidopsis (Kapulnik *et al*., 2011). Hence we investigated the root hair system in wild‐type and PDR1 OE *P. hybrida* plants. PDR1 OE seedlings had longer and denser root hairs than the wild‐type, in both main and lateral roots (Fig. 7b–k). Additionally, PDR1 OE root meristems showed root hair formation closer to the main root tip (see asterisks in Fig. 7h–k). To understand whether the root hair phenotype is caused by low nutrient conditions or whether it depends on SL redistribution as a result of *PDR1* overexpression, we tested root hair length under high‐ and low‐Pi conditions (see the Materials and Methods section). As expected, wild‐type *P. hybrida* plants had longer root hairs when grown on low Pi than on high Pi, with a significant length increase of 39.5% (Fig. S7a,b,g). *pdr1* ko mutants, on the other hand, had shorter root hairs compared with the wild‐type, independent of the nutrient conditions (Fig. S7c,d,g); however, on low Pi the root hair length still increased by 28.6%. The root hairs of PDR1 OE seedlings were long on both high‐ and low‐Pi media (Fig. S7e–g), thus showing that PDR1 is a major factor determining root hair length in *P. hybrida*.

To assess whether PDR1‐dependent SL transport may be involved in root hair elongation, we performed time‐lapse analyses using light‐sheet confocal microscopy (Maizel *et al*., 2011; Stelzer 2015; von Wangenheim *et al*., 2016) on emerging lateral roots of seedlings transgenic for *pPDR1:nls‐YFP* and *pPIN‐FORMED 1 (PIN1):nls‐RFP*, the latter involved in SL‐regulated auxin transport (Shinohara *et al*., 2013) and used here as a morphological reference for the vasculature. As *P. hybrida* roots, owing to their thickness, proved to be unsuitable for this analysis, we chose *A. thaliana* plants transgenic for the same reporters. The time‐lapse analysis (Movie S5) revealed that *pPDR1* is activated in epidermal cells as soon as root hairs elongate, and stops its activity when root hairs are fully elongated (Fig. S3h–k), implying that SL transport plays a key role during root hair elongation but not after root hairs have reached their final size. The sequential appearance of *pPDR1:nls‐YFP* and *pPIN1:nls‐RFP* (Fig. S7l,m) suggests a temporal order of hormonal action during root hair elongation.

### Endogenous changes in SL concentrations alter the auxin distribution in the root tip

**Figure 8:**
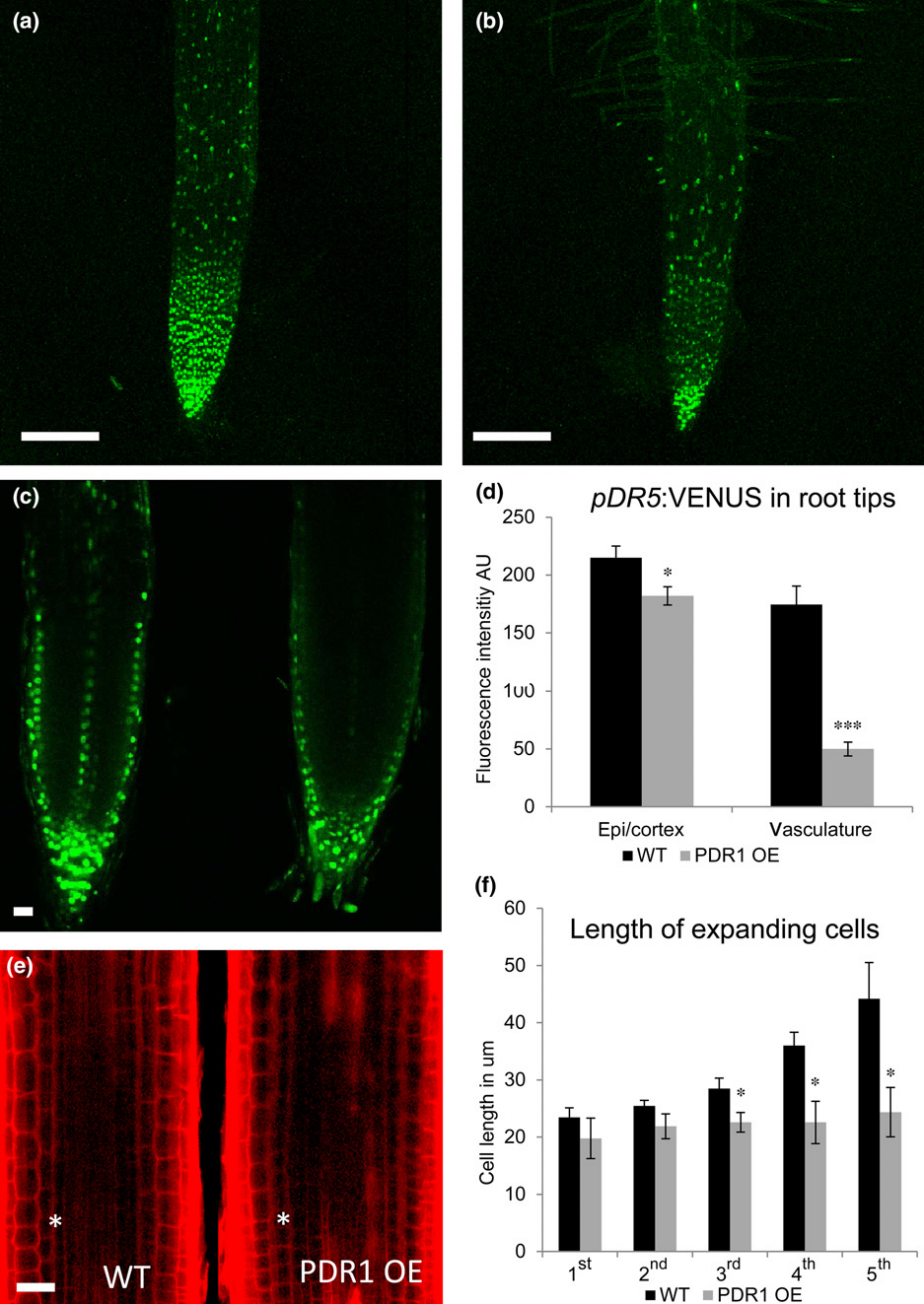
*pDR5:VENUS* patterns and cell morphology in wild‐type (WT) and PDR1 OE *Petunia hybrida* root tips. (a, b) *pDR5:VENUS* in 14‐d‐old WT (a) and PDR1 OE root (b). (c) Pattern of *pDR5:VENUS* in WT (left) and PDR1 OE root tip (right). (d) Digital quantification of *pDR5:VENUS* fluorescence in WT and PDR1 OE epidermal/cortex cells (epi/cortex) and central root vasculature (vasculature). (e) Representative propidium iodide‐stained root tips. The asterisk is located at the border between the division and the elongation zone (EZ). (f) Quantification of the cell length of the first five cells in the root tip EZ. Bars: 200 μm (a, b); 40 μm (c, e). Values are means ± SE. *, *P *<* *0.05; ***, *P *<* *0.0005.

Exogenous application of GR24 in Arabidopsis was shown to alter the endocytic recycling of the auxin transporters PIN1 and PIN2 (Shinohara *et al*., 2013; Pandya‐Kumar *et al*., 2014). Therefore, we tested whether the increased biosynthesis of endogenous SL in PDR1 OE roots might have an effect on auxin distribution. The expression pattern and intensity of the auxin reporter *pDR5:VENUS* were investigated using confocal microscopy on 2‐wk‐old seedlings grown on 1/2 MS plates. Full image stacks of *P. hybrida* root tips showed that the signal intensity of *pDR5:VENUS* was weaker in PDR1 OE than in wild‐type plants (Fig. 8a, b). The inverted fountain pattern of auxin distribution reported for Arabidopsis (Swarup & Bennett, 2003) is also present in *P. hybrida* root tips and it is weakened in PDR1 OE roots (Fig. 8c), particularly in the central vasculature (Fig. 8d), where PIN1 is expressed. Cell expansion in the elongation zone (EZ) of PDR1 OE root tips is inhibited (Fig. 8e, f), probably explaining the proximity of the first root hair to the PDR1 OE meristematic zone. These results show that endogenous changes in SL transport and biosynthesis are capable of altering auxin distribution and support the hypothesis that SL is an upstream regulator of auxin transport in the root tip (Ruyter‐Spira *et al*., 2011).

## Discussion

### SL distribution in and outside plants is regulated by *PDR1* overexpression

**Figure 9:**
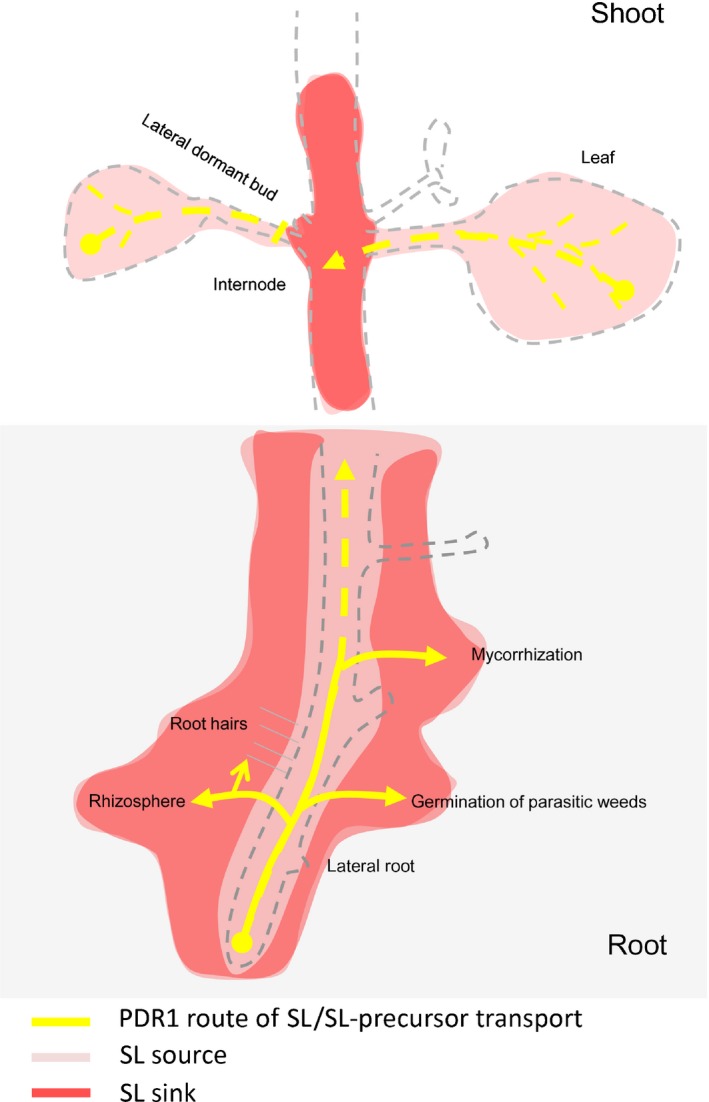
Proposed model for PDR1 routes of strigolactone (SL) transport in roots and shoots of *Petunia hybrida*. Model depicting the effects of PDR1 overexpression on SL transport in roots and shoots. PDR1 OE enhances SL exudation from the root to the rhizosphere, thus possibly reducing shoot‐ward SL transport to the stem and dampening SL concentrations in roots and leaves. As a consequence, lateral root inhibition is released, root hair elongation is induced, and mycorrhization and germination of parasitic weed seeds are enhanced. In the shoot, PDR1 drives the reallocation of SL from the leaves to the stem. This SL route is enhanced in PDR1 OE plants, thus promoting larger leaves, longer internodes than the wild‐type, and possibly playing a role in the inhibition of lateral bud outgrowth.

The overexpression of PDR1 in *P. hybrida* plants was previously reported to inhibit shoot lateral branching (Sasse *et al*., 2015). This result raised the question of whether PDR1 OE plants might increase not only SL transport but also its synthesis. Our results show that SL biosynthesis genes are induced in PDR1 OE roots and shoots, indicating that there is a cross‐regulation between transport and biosynthesis. We hypothesize that the higher amounts of SL transported from the root tip into the soil in PDR1 OE plants release *DAD1* and *MAX1* from a negative feedback regulation, which might occur in the presence of inhibitory concentrations of SL, thus allowing a higher biosynthesis of SL in shoots and roots. However, this does not necessarily cause higher SL concentrations in all tissues: SL could be even lower, probably because of simultaneously increased transport/export as seen for roots and leaves (see Fig. 9). *DAD1* also fits this model in *pdr1* ko leaves: it is strongly down‐regulated where SL accumulates. The nonresponsive behavior of *MAX1* in *pdr1 ko* leaves is probably a result of the senescence of *pdr1 ko* leaves, as *MAX1* was reported to be up‐regulated by senescence (Ueda & Kusaba, 2015). PDR1 OE stems seem to diverge from this theory, as stems are low in SL, but also in *MAX1* and *DAD1*. Still, PDR1 OE shoot lateral branching is strongly delayed (Sasse *et al*., 2015), as if, close to lateral buds, SL concentrations and/or transport are still high enough to inhibit bud outgrowth. Alternatively, the PDR1 OE‐originated redistribution of SL creates plants that are more susceptible to SL in targeted areas such as dormant buds, which might be regulated by the SL ratio between nodes/internodes rather than by the total amount of SL in the stems. It is possible that more sensitive ways of quantifying SLs would allow a finer map of SL distribution in nodes and internodes to be drawn, thus allowing us to understand if local peaks of SL synthesis and distribution are responsible for the regulation of shoot lateral branching. ABC transporters are known to be frequently induced by their substrates (Hwang *et al*., 2016); however, to our knowledge it has not yet been shown that ABC transporters affect the synthesis of their substrates similarly.

### *PDR1* overexpression differentially affects shoot tissues

As we report here in PDR1 OE plants, larger epidermal cells or rounder leaves were previously reported in *A. thaliana max2* mutants and in *ramosus‐1* (*rms*) and *rms‐2* mutants in *P. sativum* (Beveridge *et al*., 1997; Stirnberg *et al*., 2002). Impaired SL activity, either by knocking out SL receptors or biosynthetic genes, or by enhanced SL exudation into the soil, seems to affect leaf development in a similar manner. Despite the lower SL concentrations we detected in PDR1 OE leaves via the parasitic weed germination assay, PDR1 OE plants are still inhibited in lateral bud outgrowth (Sasse *et al*., 2015), a phenotype known to be mimicked by applications of GR24 on dormant buds (Gomez‐Roldan *et al*., 2008). At present, no reliable system is available to quantify SL in certain small tissues, such as lateral axils or nodes, where *pPDR1:GUS* was shown to be expressed (Kretzschmar *et al*., 2012), or in internal tissues such as the procambium, where cell division is induced by GR24 (Agusti *et al*., 2011). So we cannot track whether the SL exported from the leaf via PDR1 accumulates into the stem axils or nodes. We suggest that PDR1 overexpression has different, local effects in shoot tissues, probably because of different SL transport routes and/or different locations of the SL biosynthetic pathway. It was reported that *PhCCD7* is strongly expressed in stems (Drummond *et al*., 2015), while *PhMAX1*,* AtD14* and *AtMAX1* (Booker *et al*., 2005; Drummond *et al*., 2011; Chevalier *et al*., 2014) are also present in leaves. The SL source and sink map therefore appears to be tissue‐specific; based on our results, we propose the leaf‐to‐stem route as a new SL transport route that is important in the regulation of SL concentrations in leaves and stems. The function of this route seems to be the regulation of leaf senescence, which is SL‐dependent (Ueda & Kusaba, 2015), but it might also contribute to the SL‐driven inhibition of lateral bud growth.

### *PDR1* overexpression primes plants to starvation via the enhanced root system

PDR1 OE plants grown in full nutrient conditions show several morphological traits common to plants grown in low‐Pi conditions, such as inhibition of lateral shoot growth, induction of lateral roots and root hair development (Zhang *et al*., 2014a,b). We propose that PDR1 overexpression‐induced synthesis of SL triggers a plant response similar to phosphate starvation, and hence results in plants that are primed to starvation before they experience it. Such behavior could explain why PDR1 OE plants can sequestrate more phosphate from soils, and mycorrhize and produce biomass faster than the wild‐type only in phosphate conditions lower than in full soil, conditions in which wild‐type plants need more time to adapt their architecture to the challenging environment. The observed PDR1 overexpression‐dependent increase in lateral root number particularly affects shallow lateral roots. The topsoil commonly shows higher nutrient (and particularly P) concentrations. Pi resources are limited as well as expensive to explore (Cordell *et al*., 2009). Therefore plants with higher capacities for Pi uptake are of agricultural interest. Additional field tests will be necessary to obtain a broader view of which conditions and which soils confer a similar advantage to PDR1 OE plants as that seen in glasshouse tests.

Our results indicate that root hair formation is strongly dependent on exuded SL and/or the presence of active transport of SL by PDR1 through the epidermal layer. Our time‐lapse analyses showed that PDR1 is active from initiation to full elongation of each root hair. As SL was reported to regulate cytoskeletal dynamics (Pandya‐Kumar *et al*., 2014), we suggest that the extra SL transported by PDR1 allows an extended development of root hairs. We observed a reduced SL concentration within PDR1 OE roots, while *pdr1* mutants, which have shorter root hairs compared with the wild‐type, were shown not to differ from the latter in root internal SL concentrations (Kretzschmar *et al*., 2012). These observations indicate that the PDR1‐triggered SL release into the rhizosphere probably has a stronger impact on root hair formation than does internal SL redistribution.

### *PDR1* overexpression affects the crosstalk between SLs and auxins

The crosstalk between SL and auxins is altered in PDR1 OE plants and thus exerts a role in shaping PDR1 OE plant phenotypes. GR24 treatments cause the removal of the auxin carrier PIN1 from the plasma membrane (Shinohara *et al*., 2013) and PIN1 protein abundances are down‐regulated in PDR1 OE root tips (Sasse *et al*., 2015). On the other hand, the auxin carrier PIN2 is positively regulated by SL (Pandya‐Kumar *et al*., 2014; Sasse *et al*., 2015). Also, the expression of *DAD1* is negatively regulated by SL but positively regulated by auxins (Hayward *et al*., 2009). Auxin transport/allocation could be influenced in several tissues by PDR1 overexpression, thus also changing *DAD1* expression and consequently SL biosynthesis. Analyses of the auxin patterns in PDR1 OE root tips showed that changes in endogenous SL concentrations can alter auxin transport and patterning, thus affecting cell length in the EZ and consequently root hair density close to the root tip. Several *pin* mutants were indeed reported to inhibit cell elongation in the EZ (Blilou *et al*., 2005). In root tips SL might influence the abundances of several PIN proteins at the plasma membrane either directly or by acting on auxin flows directed by PIN1 and PIN2. Additional auxin and PIN quantifications in specific plant tissues such as root hairs, lateral roots, lateral buds and leaves might elucidate in detail the mechanisms behind the crosstalk between SL and auxin transporters. Based on the results shown here, we propose that SL regulates cell expansion by changing the efficiency of auxin transport: this change reduces cell expansion (as seen in the EZ of PDR1 OE root tips) or allows cell expansion (as seen in leaf epidermal cells of PDR1 OE plants).

### Applications of *PDR1* overexpression

Despite the costs of producing a larger root system, PDR1 OE plants are still able to produce more shoot biomass when grown on soils that are suboptimal for wild‐type plants. The morphological changes in the root system architecture of PDR1 OE reported here show that many of the phenotypes observed when GR24 is exogenously applied, such as root hair elongation and lateral root induction on low Pi soil, can also be obtained simply by overexpressing the SL transporter, even independently of the soil nutrient conditions. A critical point about exploiting the PDR1 overexpression strategy for field‐grown plants is the possible presence of parasitic weed seeds in some soils and regions (Parker, 2012). Also, a tradeoff with PDR1 OE‐increased biomass production might be a higher sensitivity to drought and salinity stress: SL is reported to induce drought and salinity tolerance in Arabidopsis (Ha *et al*., 2014). We have preliminary data suggesting that PDR1 OE‐expanded leaf blade might cause higher transpiration in water‐limited conditions compared with the wild‐type. However, mycorrhization is known to alleviate drought stress (Ruiz‐Lozano *et al*., 2016) and the increased exudation of SLs from PDR1 OE roots induces higher mycorrhization levels than in the wild‐type, which might balance drought and salinity sensitivity. On the other hand, SL‐driven approaches have also been shown to be effective against parasitic weeds, such as suicidal germination, which promotes the germination of parasitic weeds in the absence of host plants (Kgosi *et al*., 2012; Khosla & Nelson, 2016). Besides, a PDR1 overexpression strategy could provide a solution to improve plant nutrition for crops that are not hosts for parasitic weeds or grown on fields without parasites. PDR1 overexpression may also be combined with two approaches that have already been proposed: overproduction of citrate (Lopez‐Bucio *et al*., 2000), which was shown to have a positive effect on phosphate nutrition; and/or overexpression of ABCG37/PDR9 (Fourcroy *et al*., 2014), which was reported to exhibit a positive effect on iron nutrition via coumarin exudation.

In summary, our studies shed new light on SL transport routes and targets. These could provide a solution for improving plant nutrition and be a strategy for sustainable agriculture on low‐Pi soils, where an increase in the root system volume and/or the symbiosis with mycorrhizal fungi is required to allow the plant to exploit larger soil volumes. Furthermore, screening for accessions with high PDR1 expression could be a new approach to isolate plant varieties with higher mycorrhization efficiency and improved root system architecture.

## Author contributions

L.B., G.L. and E.M. conceived and designed research. G.L., L.B., J.P., R.d.B.F., A.E., C.G., M.S., O.H., J.S., C.M. conducted experiments. L.B., G.L. R.d.B.F. and E.M. analyzed data. L.B., G.L., J.P., R.d.B.F., A.W., E.S. and E.M. wrote the manuscript. All authors read and approved the manuscript.

## Supporting information

Please note: Wiley Blackwell are not responsible for the content or functionality of any Supporting Information supplied by the authors. Any queries (other than missing material) should be directed to the *New Phytologist* Central Office.

**Fig. S1** Mycorrhization quantifications via the grid method and gene expression analyses on *PhPT3* and *PhPT5* in *Petunia hybrida* roots.
**Fig. S2** PDR1 OE effects on SL biosynthesis and P uptake in *Petunia hybrida*.
**Fig. S3** Semiquantitative SL quantification, ^3^H‐GR24 transport and leaf senescence in wild‐type and *pdr1* ko *Petunia hybrida* leaves.
**Fig. S4** GR24 transport quantification in wild‐type, PDR1 OE and *pdr1* ko *Petunia hybrida* leaves (Time0 = loading time; Time1 = export time).
**Fig. S5** Nonfluorescent Chl catabolite (NCC) quantification in wild‐type and PDR1 OE leaves from 3‐month‐old *Petunia hybrida* plants.
**Fig. S6** X‐ray computed tomography on roots of 6‐wk‐old *Petunia hybrida* plants.
**Fig. S7** The influence of PDR1 on root hair elongation in *Petunia hybrida*.
**Table S1** *P*‐ and *n*‐values for Student's *t*‐test statistical analyses
**Table S2** Parameters for X‐ray computed tomography on *Petunia hybrida* roots
**Methods S1** Supplementary material and methods.

**Movie S1** X‐ray computed tomography multiscan of 42‐d‐old wild‐type *Petunia hybrida* roots grown on clay+.

**Movie S2** X‐ray computed tomography multiscan of 42‐d‐old PDR1 OE *Petunia hybrida* roots grown on clay+.

**Movie S3** X‐ray computed tomography multiscan of 60‐d‐old wild‐type *Petunia hybrida* roots grown on clay+.

**Movie S4** X‐ray computed tomography multiscan of 60‐d‐old PDR1 OE *Petunia hybrida* roots grown on clay+.

**Movie S5** Time lapse (35 h) of a developing lateral root from *Arabidopsis thaliana* acquired via light sheet fluorescence microscopy.
