# Supplementary material for: Changes in the allocation of endogenous strigolactone improve plant biomass production on phosphate‐poor soils
Source: New Phytol. 2017 Oct 30;217(2):784–98. doi: 10.1111/nph.14847 (PMC5765447; doi:10.1111/nph.14847)
Supplement: Supplementary file 1 — Fig. S1 Mycorrhization quantifications via the grid method and gene expression analyses on PhPT3 and PhPT5 in Petunia hybrida roots. Fig. S2 PDR1 OE effects on SL biosynthesis and P uptake in Petunia hybrida. Fig. S3 Semiquantitative SL quantification, 3H‐GR24 transport and leaf senescence in wild‐type and pdr1 ko Petunia hybrida leaves. Fig. S4 GR24 transport quantification in wild‐type, PDR1 OE and pdr1 ko Petunia hybrida leaves (Time0 = loading time; Time1 = export time). Fig. S5 Nonfluorescent Chl catabolite (NCC) quantification in wild‐type and PDR1 OE leaves from 3‐month‐old Petunia hybrida plants. Fig. S6 X‐ray computed tomography on roots of 6‐wk‐old Petunia hybrida plants. Fig. S7 The influence of PDR1 on root hair elongation in Petunia hybrida. Table S1 P‐ and n‐values for Student's t‐test statistical analyses Table S2 Parameters for X‐ray computed tomography on Petunia hybrida roots Methods S1 Supplementary material and methods. [file NPH-217-784-s001.pdf]

New Phytologist

New Phytologist Supporting Information

Article title: Changes in the allocation of endogenous strigolactone improve plant biomass production on phosphate-poor soils
Authors: Guowei Liu, Johannes Pfeifer, Rita de Brito Francisco, Aurelia Emonet, Marina Stirnemann, Christian Gübeli, Olivier Hutter, Joëlle Sasse, Christian Mattheyer, Ernst Stelzer, Achim Walter, Enrico Martinoia and Lorenzo Borghi
Article acceptance date: 05 September 2017

The following Supporting Information is available for this article:

Fig. S1 Mycorrhization quantifications via the grid method and gene expression analyses on *PhPT3* and *PhPT5* in P*etunia hybrida* roots.

Fig. S2 PDR1 OE effects on SL biosynthesis and P uptake in *Petunia hybrida*.

Fig. S3 Semi-quantitative SL quantification, ^{3}H-GR24 transport and leaf senescence in wildtype and *pdr1* ko *Petunia hybrida* leaves.

Fig. S4 GR24 transport quantification in wildtype, PDR1 OE and *pdr1* ko *Petunia hybrida* leaves (Time0 = loading time; Time1= export time).

Fig. S5 Non-fluorescent chlorophyll catabolite (NCC) quantification in wildtype and PDR1 OE *Petunia hybrida* leaves from 3 month old plants.

Fig. S6 X-ray computed tomography on roots of 6 week-old *Petunia hybrida* plants.

Fig. S7 The influence of PDR1 on root hair elongation in *Petunia hybrida*.

Table S1 *p* and *n* values for Student's t-test statistical analyses.

Table S2 Parameters for X-ray computed tomography on *Petunia hybrida* roots**.**

Methods S1 Supplementary material and methods.

Movie S1 X-ray computed tomography multiscan of 42 d-old wildtype *Petunia hybrida* roots grown on clay+. 360° rotation recorded on its y-axis

Movie S2 X-ray computed tomography multiscan of 42 d-old PDR1 OE *Petunia hybrida* roots grown on clay+. 360° rotation recorded on its y-axis

Movie S3 X-ray computed tomography multiscan of 60 d-old wildtype *Petunia hybrida* roots grown on clay+. 360° rotation recorded on its y-axis

Movie S4 X-ray computed tomography multiscan of 60 d-old PDR1 OE multiscan of 60 d-old wildtype *Petunia hybrida* roots grown on clay+. 360° rotation recorded on its y-axis

Movie S5 35 h-long time-lapse of a developing lateral root from *Arabidopsis thaliana* acquired via light sheet fluorescence microscopy. The examined plant is transgenic for *pPDR1:nls-YFP* and *pPIN1:nls-RFP*

Fig. S1 (a) Mycorrhization rates on clay+. (b) Mycorrhization rates on natural soil mix. (c-j) Representative pictures of mycorrhization events in petunia roots. (k-l) *PhPT3* and *PhPT5* (m-n) expression levels 6 and 8 w.a.g in petunia grown on natural soil mix and clay+. Bars, 100 μm. Values are mean +/- SE. * = p < 0.05; ** = p < 0.005; *** = p < 0.0005

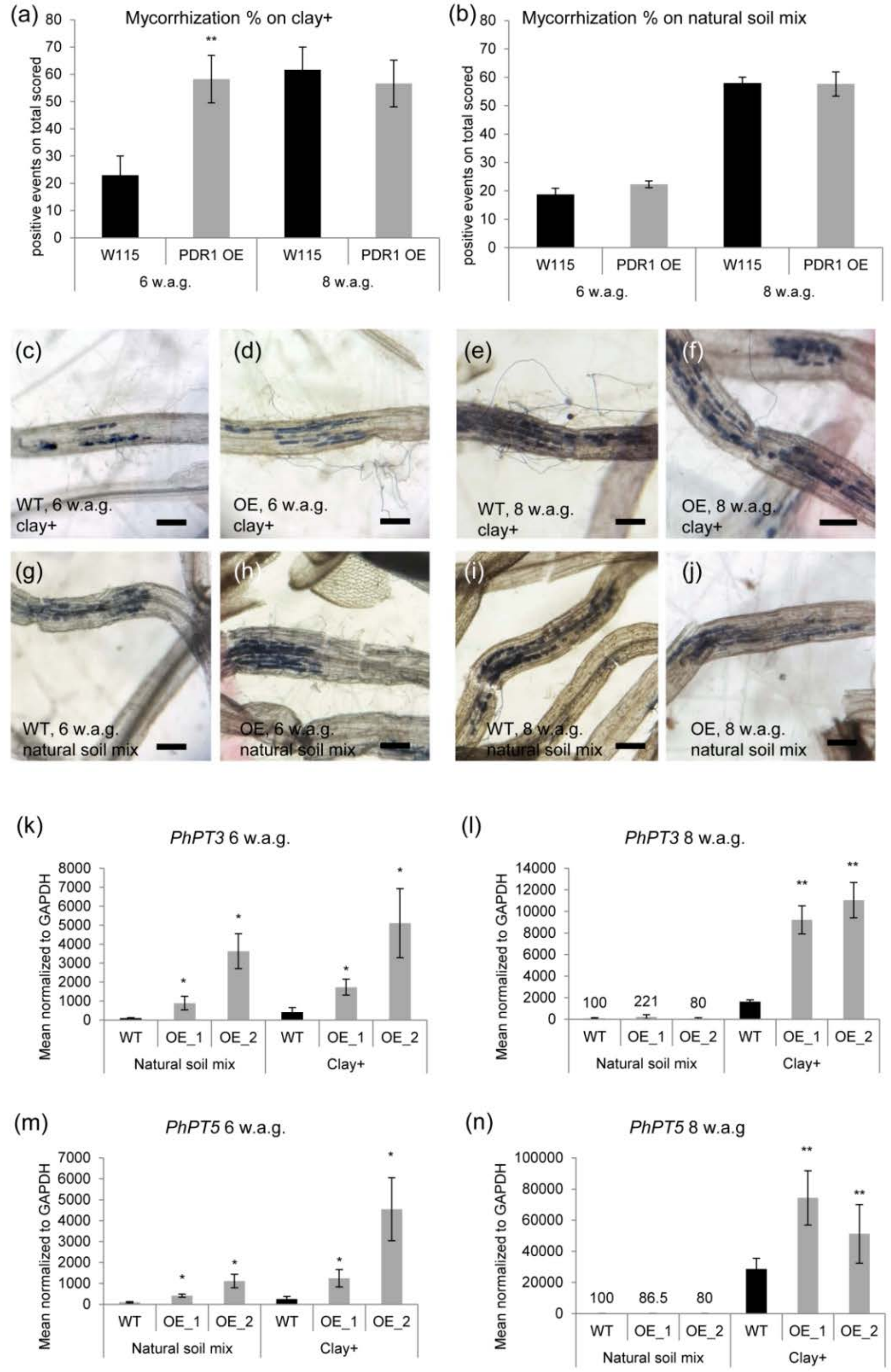

Fig. S2 (a, b) *Phelipanche ramosa* germination induction via 1 nm or 1 um GR24 challenged with shoot extracts from Petunia PDR1 OE, *pdr1 ko* and wildtype extracts. (c) Ratios of P quantification in petunia wildtype and PDR1 OE plants. (d-f) Gene expression levels in WT and 3 different PDR1 OE lines of *PDR1*, *DAD1* and *MAX1.* Values are mean +/- SE. * = p < 0.05; ** = p < 0.005; *** = p < 0.0005

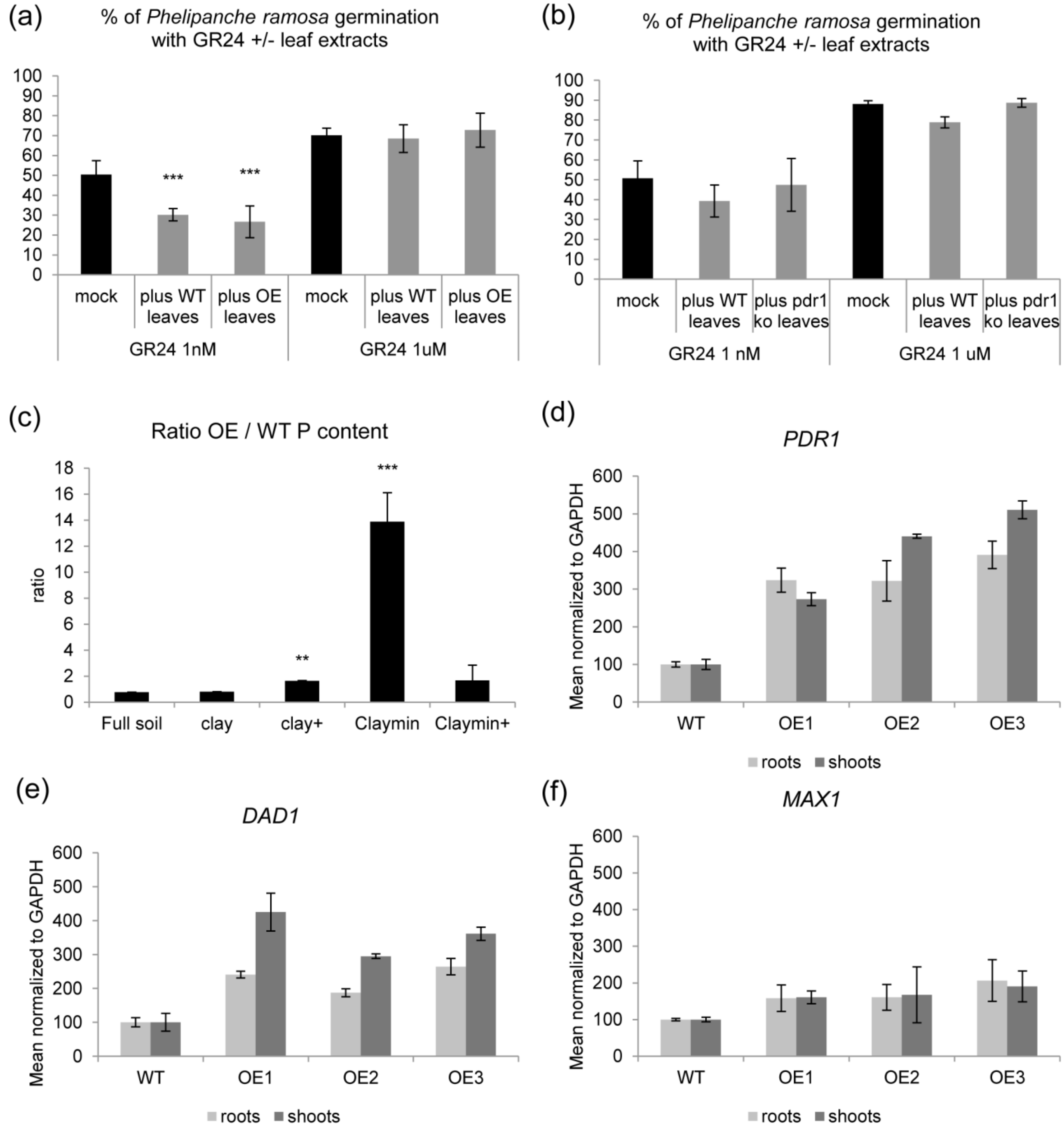

Fig. S3 (a) Germination rate of *Phelipanche ramosa* seeds with leaf extracts of wildtype and *pdr1* ko plants. (b) Decays per minute (DPM) of ^{3}H-GR24 present in *pdr1* ko unloaded sap relative to ^{3}H-GR24 leaf content and (c) to ^3H_2O leaf content. (d) Senescence-related leaf phenotypes in wildtype and in *pdr1* ko plants from the leaf -6 grown before the transition to flowering time down to leaf -14. (e) Leaf area in wildtype and *pdr1 ko* plants. (f) Gene expression levels of petunia *MAX1* and *DAD1* in leaves -11/-14. (g) Gene expression levels of petunia *ORE1-like*, *SAG12-like* and *SAG13-like* in leaves -11/-14. Bars, 2 cm. Values are mean +/- SE. * = p < 0.05; ** = p < 0.005; *** = p < 0.0005

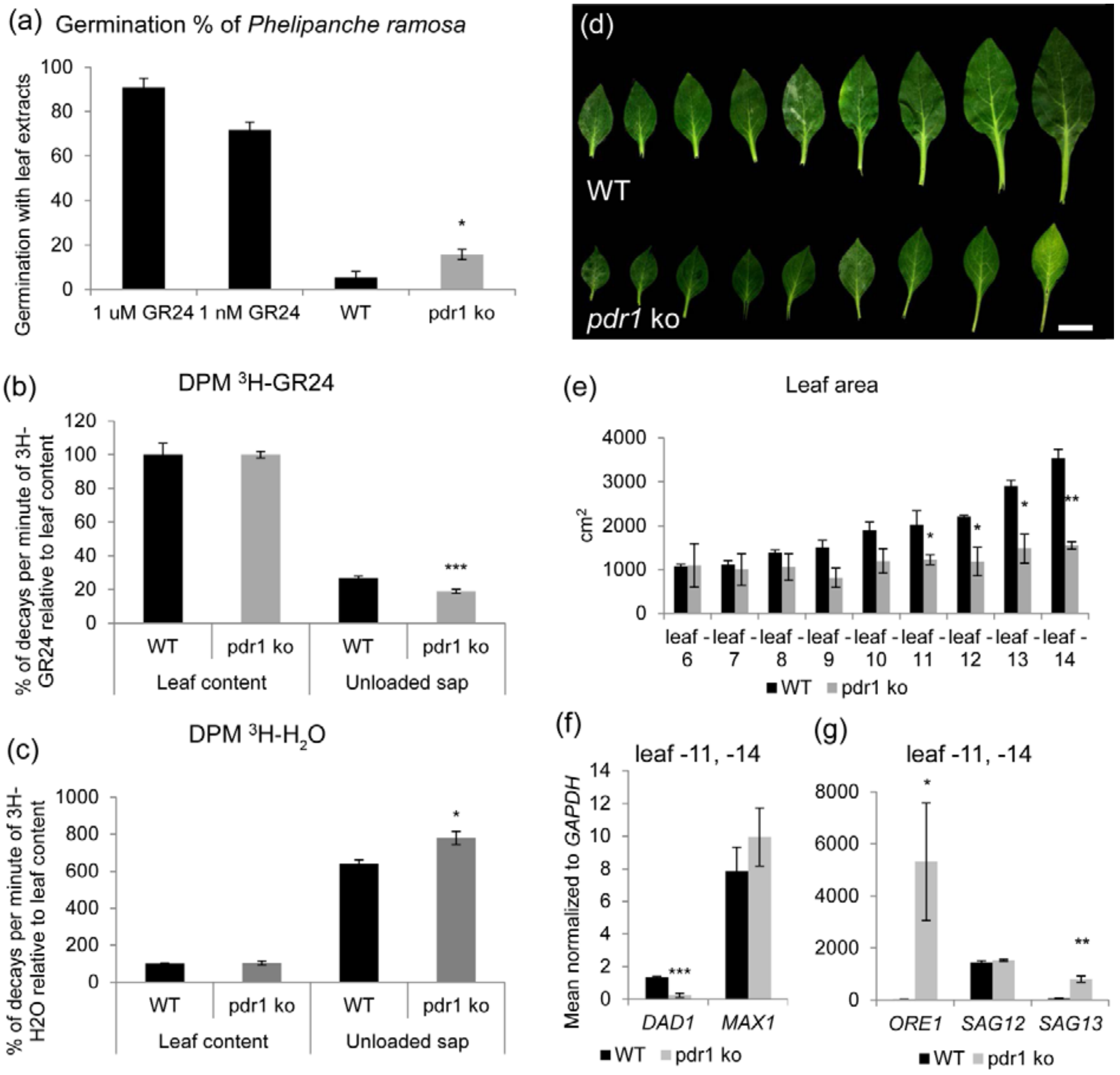

Fig. S4 (a) ng of transported GR24 in wildtype and PRD1 OE leaves. (b) ng of transported GR24 in wildtype and *pdr1* ko leaves. (c) Extracted Ion Chromatogram (EIC) and MS/MS (299>97.02) spectra of GR24 standard. (d) MS and MS/MS spectra of wildtype leaf exudate. Red arrow highlights GR24 $[M+H]^+$. Blue diamond indicates precursor ion. Values are mean +/- SE. * = $p < 0.05$; ** = $p < 0.005$; *** = $p < 0.0005$

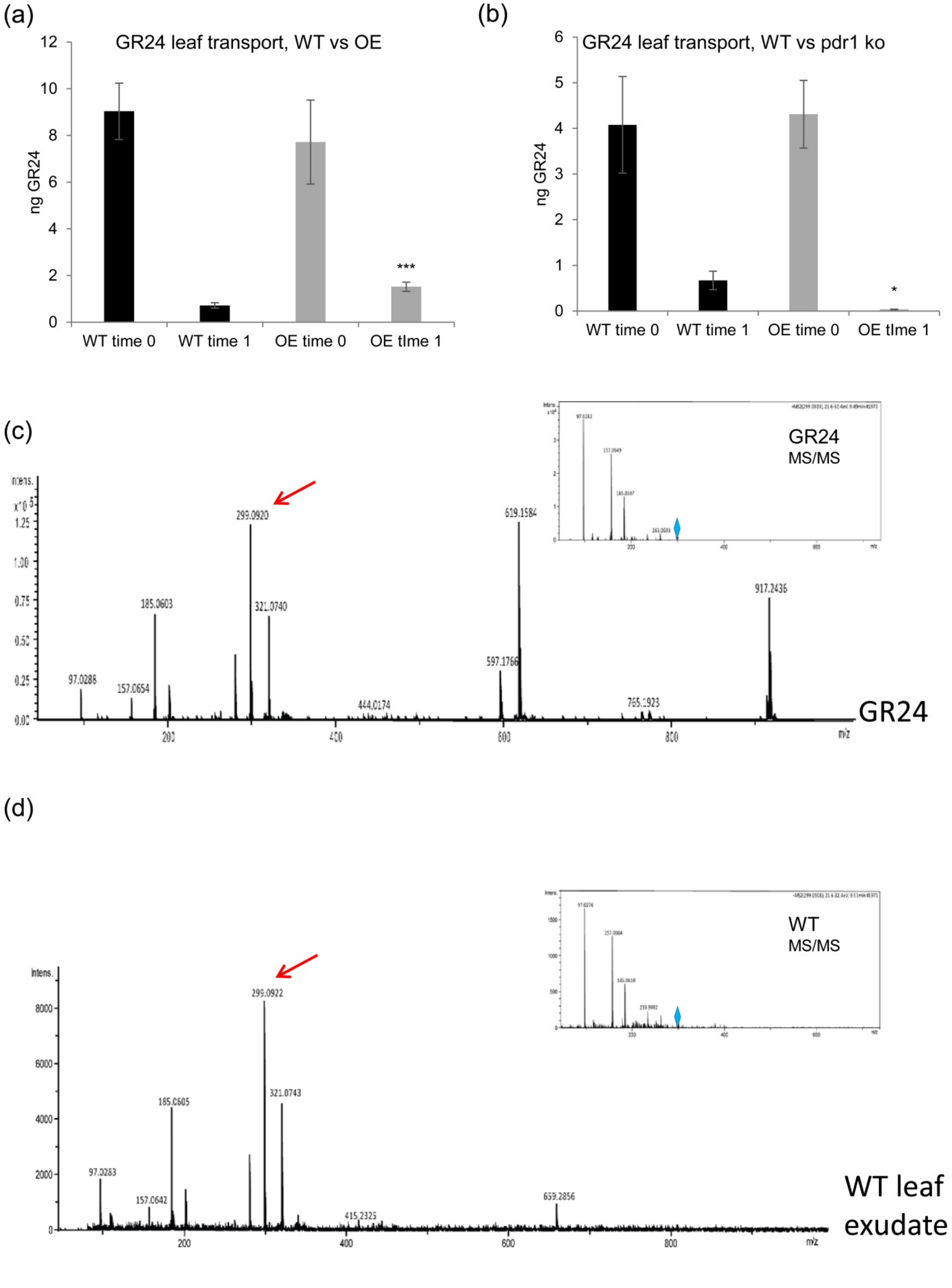
(a)
GR24 leaf transport, WT vs OE
ng GR24
12
10
8
6
4
2
0
***
WT time 0
WT time 1
OE time 0
OE tIme 1
(b)
GR24 leaf transport, WT vs pdr1 ko
ng GR24
6
5
4
3
2
1
0
*
WT time 0
WT time 1
OE time 0
OE tIme 1
(c)
Intens.
1.25
1.00
0.75
0.50
0.25
0.00
299.0920
619.1584
185.0603
321.0740
917.2436
97.0288
157.0654
444.0174
597.1766
765.1923
200
400
600
800
m/z
GR24
GR24
MS/MS
(d)
Intens.
8000
6000
4000
2000
0
299.0922
185.0605
321.0743
97.0283
157.0642
415.2325
659.2856
200
400
600
800
m/z
WT leaf
exudate
WT
MS/MS

Fig. S5 Non-fluorescent chlorophyll catabolites (NCC) quantifications from leaves of 3 month old *Petunia hybrida* plants. (a) NCC_806. (b) NCC_892. (c) EIC of NCC_806 ($C_{41}H_{50}N_4O_{13}$) and NCC_892 ($C_{44}H_{52}N_4O_{16}$) of wildtype leaves. (d) EIC of NCC_806 and NCC_892 of PRD1 OE leaf extracts. Red arrow highlights $[M+H]^+$ of the identified chlorophyll catabolites. Values are mean +/- SE. * = $p < 0.05$; ** = $p < 0.005$; *** = $p < 0.0005$

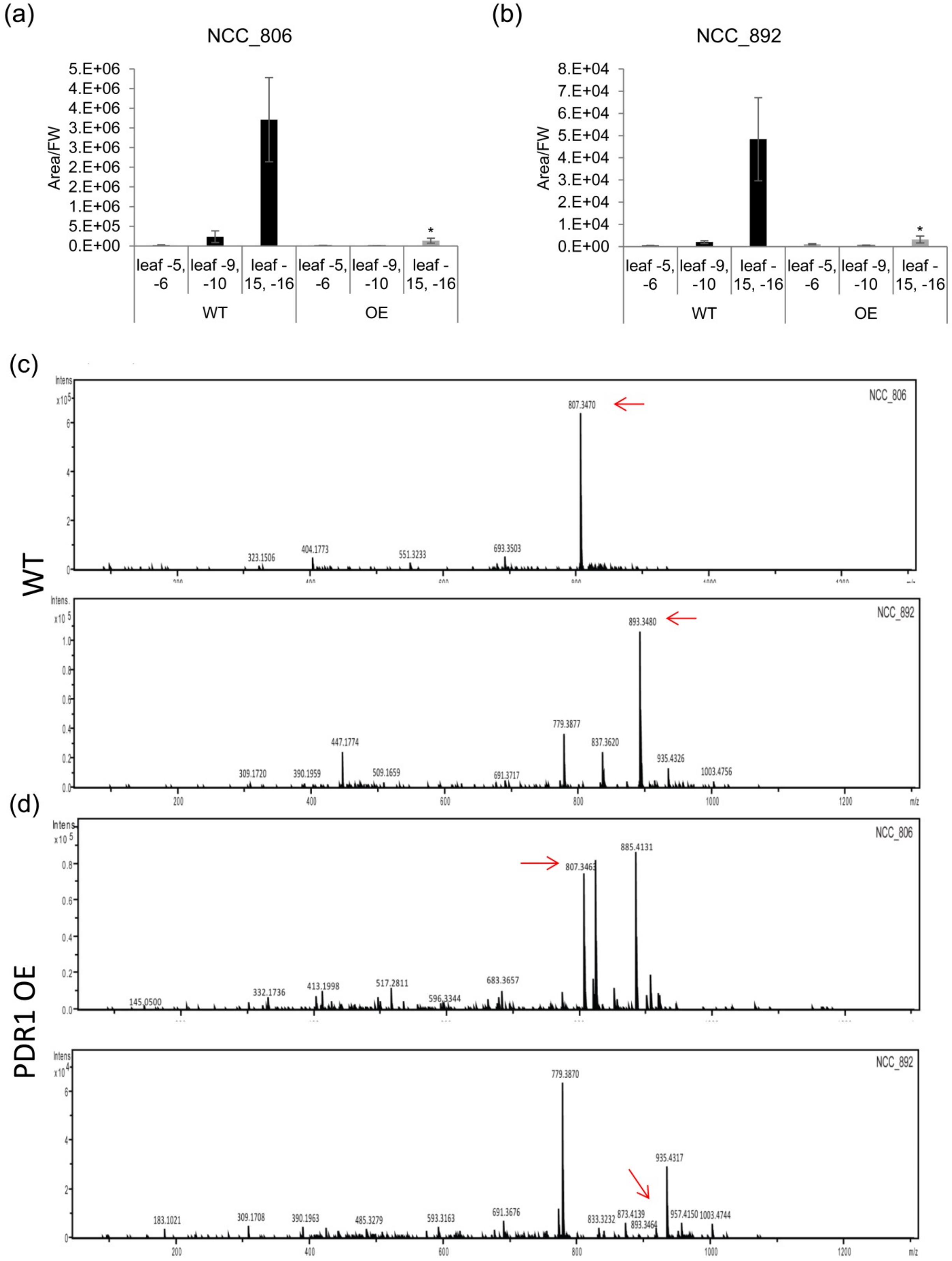
(a)
NCC_806
Area/FW
5.E+06
4.E+06
4.E+06
3.E+06
3.E+06
2.E+06
2.E+06
1.E+06
5.E+05
0.E+00
leaf -5, -6
leaf -9, -10
leaf -15, -16
WT
OE
*
(b)
NCC_892
Area/FW
8.E+04
7.E+04
6.E+04
5.E+04
4.E+04
3.E+04
2.E+04
1.E+04
0.E+00
leaf -5, -6
leaf -9, -10
leaf -15, -16
WT
OE
*
(c)
WT
Intens. x10^5
NCC_806
807.3470
323.1506
404.1773
551.3233
693.3503
NCC_892
893.3480
447.1774
779.3877
837.3620
935.4326
309.1720
390.1959
509.1659
691.3717
1003.4756
200
400
600
800
1000
1200
m/z
(d)
PDR1 OE
NCC_806
807.3463
885.4131
145.0500
332.1736
413.1998
517.2811
596.3344
683.3657
Intens. x10^4
NCC_892
779.3870
935.4317
183.1021
309.1708
390.1963
485.3279
593.3163
691.3676
833.3232
873.4139
893.3464
957.4150
1003.4744

Fig. S6 (a) Heat map (root thickness) of 6-wk-old wildtype (left) and PDR1 OE (right) roots grown on clay+ (see also Movie S1, S2). (b) Quantification of lateral roots in WT and PDR1 OE roots +/- mycorrhizal inoculum (myc). (c) Ratio between PDR1 OE and WT root surfaces and volumes +/- myc. Bars, 1.5 cm. Values are mean +/- SE.

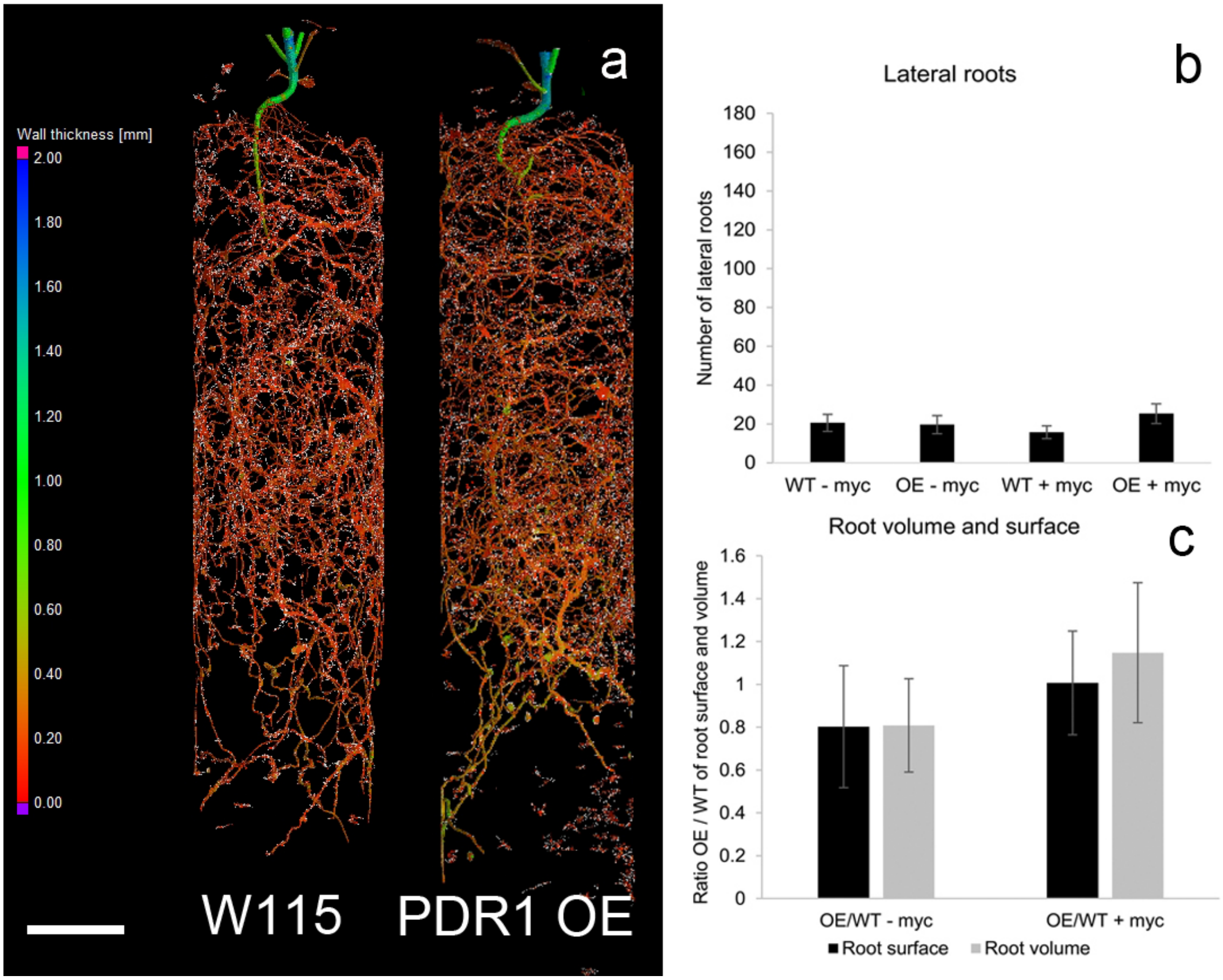

Fig. S7 The influence of PDR1 on root hair elongation. Root hair length responsiveness to different Pi concentrations in wildtype and PDR1 OE Petunia seedlings. (a-f) Representative lateral roots emerged after transferring 3-week-old petunia seedlings from ½ MS plates to mock (a, c, e) or low phosphate (b, d, f) agar plates. (a, b) Wildtype. (c, d) pdr1 ko. (e, f) PDR1 OE. (g) Quantification of root hair length in different Pi conditions. (h-k) Still frames from a time lapse (see Movie S5) of pPDR1:nls-YFP; pPIN1:nls-RFP during lateral root development in Arabidopsis thaliana seedlings. Arrowheads: pPDR1 is active in elongating (h, i) but not in elongated root hairs (j, k). (l) Still frame from time point 35 h of Movie S5 and root hairs tagged from "a" to "o" for tracking in m. (m) Temporal quantification of pPDR1:nls-YFP (yellow bars) and pPIN1:nls-RFP (red bars) signals in root hairs from 0 to 35 hours after beginning of the time lapse. pPDR1:nls-YFP activity is present at root hair initiation and disappears at full elongation, eventually substituted by pPIN1:RFP. Grey bars represent the situation when no nucleus in epidermal cells is visible. Bars, 400 μm. Values are mean +/- SE. * = $p < 0.05$; ** = $p < 0.005$; *** = $p < 0.0005$

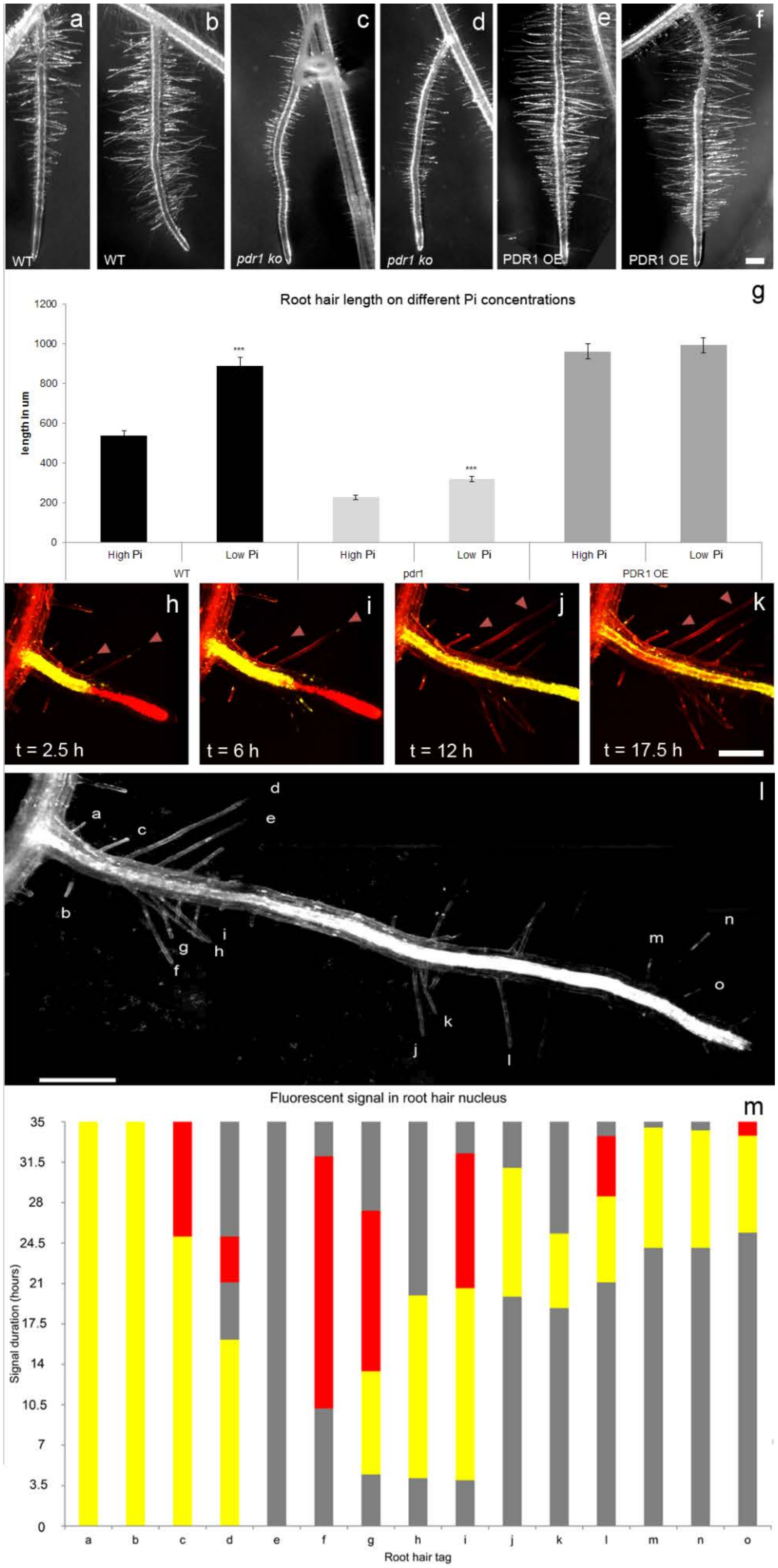

a
b
c
d
e
f
WT
WT
pdr1 ko
pdr1 ko
PDR1 OE
PDR1 OE
g
Root hair length on different Pi concentrations
length in um
1200
1000
800
600
400
200
0
***
***
High Pi
Low Pi
High Pi
Low Pi
High Pi
Low Pi
WT
pdr1
PDR1 OE
h
i
j
k
t = 2.5 h
t = 6 h
t = 12 h
t = 17.5 h
l
a
b
c
d
e
f
g
h
i
j
k
l
m
n
o
m
Fluorescent signal in root hair nucleus
Signal duration (hours)
35
31.5
28
24.5
21
17.5
14
10.5
7
3.5
0
a b c d e f g h i j k l m n o
Root hair tag

Table S1 p and n values for Student's t-test statistical analyses.

Figure panel	Figure	Significance	*p value*	n
Figure 1	a	W115 to PDR1 OE	1.14E-02	4
		W115xW138 to *pdr1* ko	8.00E-04	4
	b	soil to clay	4.80E-02	6
		clay to mineral soil	1.90E-02	6
	c	OE_2 to WT on natural soil mix	7.12E-03	4
		OE_1 to WT on clay+	2.75E-02	3
		OE_2 to WT on clay+	4.31E-03	3
	d	OE_1 to WT on clay+	8.72E-04	4
		OE_2 to WT on clay+	6.59E-04	4
	e	OE_2 to WT on natural soil mix	6.09E-03	4
		OE_2 to WT on clay+	4.81E-02	4
	f	OE_1 to WT on clay+	5.48E-05	4
		OE_2 to WT on clay+	4.27E-03	3
	g	W115 to PDR1 OE 6 w.a.g.	3.10E-03	8
		W115 to PDR1 OE 8 w.a.g.	1.00E-04	8
	h	W115 to PDR1 OE	1.90E-03	8
Figure 2	c	W115 to PDR1 OE root	2.44E-02	4
		W115 to PDR1 OE shoot	2.03E-03	4
Figure 3	b	W115 to PDR1 OE l5	4.33E-02	3
		W115 to PDR1 OE l6	4.45E-02	3
		W115 to PDR1 OE l7	3.26E-02	3
		W115 to PDR1 OE l8	5.70E-03	3
		W115 to PDR1 OE l9	5.90E-03	3
		W115 to PDR1 OE l10	6.00E-04	3
		W115 to PDR1 OE l11	7.00E-04	3
		W115 to PDR1 OE l12	4.39E-02	3
	d	W115 to PDR1 OE in7	1.03E-02	3
		W115 to PDR1 OE in8	1.70E-03	3
		W115 to PDR1 OE in9	8.70E-03	3
	e	W115 to PDR1 OE	5.26E-06	16
	g	W115 to PDR1 OE	2.54E-12	40
		W115 to PDR1 OE	4.19E-18	40
	h	W115 to PDR1 OE	3.90E-02	40
	i	W115 to PDR1 OE myc- in2	3.80E-01	5
		W115 to PDR1 OE myc- in3	3.62E-01	5
		W115 to PDR1 OE myc- in4	6.13E-01	5
		W115 to PDR1 OE myc- in5	6.05E-01	5

		W115 to PDR1 OE myc- in6	3.90E-03	5
		W115 to PDR1 OE myc- in7	6.60E-03	5
		W115 to PDR1 OE myc- in8	5.40E-03	5
		W115 to PDR1 OE myc+ in2	7.08E-01	5
		W115 to PDR1 OE myc+ in3	4.79E-02	5
		W115 to PDR1 OE myc+ in4	6.71E-05	5
		W115 to PDR1 OE myc+ in5	2.20E-02	5
		W115 to PDR1 OE myc+ in6	9.90E-03	5
		W115 to PDR1 OE myc+ in7	4.69E-02	5
		W115 to PDR1 OE myc+ in8	3.66E-02	5
	j	W115 to PDR1 OE High	1.10E-03	40
		W115 to PDR1 OE Low	2.00E-04	40
Figure 4	e	W115 to PDR1 OE shoot on clay+	4.20E-03	4
		W115 to PDR1 OE shoot on Claymin	9.09E-03	4
	f	W115 to PDR1 OE root on clay+	2.44E-02	4
		W115 to PDR1 OE root on Claymin	1.02E-02	4
	g	W115 to PDR1 OE root	2.00E-03	3
		W115 to PDR1 OE shoot	4.80E-02	3
	h	W115 to PDR1 OE root	7.00E-03	3
		W115 to PDR1 OE shoot	4.60E-02	3
	i	W115 to PDR1 OE root	4.60E-02	3
		W115 to PDR1 OE shoot	6.70E-03	3
	j	MAX1, WT to PDR1 OE leaves	3.06E-04	3
		MAX1, WT to PDR1 OE stem	4.40E-03	3
		DAD1, WT to PDR1 OE stem	9.45E-05	3
Figure 5	b	W115 to PDR1 OE	4.70E-04	4
	e	W115 to PDR1 OE	2.30E-04	4
		W115 to PDR1 OE	3.10E-04	4
	f	W115 to PDR1 OE	4.50E-05	4
		W115 to PDR1 OE	1.20E-03	4
	g	W115 to PDR1 OE	1.50E-06	4
		W115 to PDR1 OE	5.60E-03	4
Figure 6	b	W115 to PDR1 OE - myc	2.02E-02	4
		W115 to PDR1 OE + myc	4.00E-04	4
	c	W115 to PDR1 OE	4.70E-03	4
		W115 to PDR1 OE	1.57E-02	4
Figure 7	a	W115 to PDR1 OE	1.06E-02	14
	b	W115 to PDR1 OE	1.33E-02	4
	c	W115 to PDR1 OE	1.46E-18	50
Figure 8	d	W115 to PDR1 OE epi/cortex	1.55E-02	38
		W115 to PDR1 OE vasculature	1.43E-06	38
	f	W115 to PDR1 OE 3rd	1.43E-02	35
		W115 to PDR1 OE 4th	8.08E-03	35

		W115 to PDR1 OE 5th	7.55E-03	35
Figure S1	a	6 w.a.g., W115 vs PDR1 OE	3.60E-03	8
	k	Nat soil mix, WT vs OE1	4.09E-02	4
		Nat soil mix, WT vs OE2	1.76E-02	4
		Clay+, WT vs OE1	4.87E-02	4
		Clay+, WT vs OE2	4.33E-02	4
	l	Clay+, WT vs OE1	4.09E-03	4
		Clay+, WT vs OE2	4.46E-03	4
	m	Nat soil mix, WT vs OE1	1.43E-02	4
		Nat soil mix, WT vs OE2	1.84E-02	4
		Clay+, WT vs OE1	3.96E-02	4
		Clay+, WT vs OE2	2.50E-02	4
	n	Clay+, WT vs OE1	2.38E-03	4
		Clay+, WT vs OE2	3.67E-03	4
Figure S2	a	mock to plus WT leaves	2.09E-04	8
		mock to plus OE leaves	3.89E-04	8
	c	clay+	1.90E-03	5
		Claymin	2.00E-04	5
Figure S3	a	WT vs pdr1 ko	3.00E-02	8
	b	WT vs pdr1 ko	4.50E-04	6
	c	WT vs pdr1 ko	2.19E-02	6
	e	WT vs pdr1 ko	4.32E-02	3
		WT vs pdr1 ko	4.13E-02	3
		WT vs pdr1 ko	2.28E-02	3
		WT vs pdr1 ko	2.76E-03	3
	f	DAD1 WT to pdr1 ko	1.18E-03	4
	g	ORE1-like WT to pdr1 ko	2.34E-02	4
		SAG13-like WT to pdr1 ko	3.05E-03	4
Figure S4	a	WT to PDR1 OE	5.66E-03	6
	b	WT to pdr1 ko	2.66E-02	6
Figure S5	a	WT vs PDR1 OE	3.31E-02	6
	b	WT vs PDR1 OE	3.32E-02	6
Figure S7	g	WT High P to Low P	2.27E-09	35
		pdr1 ko High P to Low P	2.09E-06	35

Table S2 Parameters for X-ray computed tomography.

Table 1 Sample and acquisition parameters for X-ray computed tomography

	Experiment 1	Experiment 2	Experiment 3
**Cultivation parameters**			
Cylinder internal diameter [cm]	6	6	3.4
Mycorrhiza inoculation	yes	no	yes and no
Plant age [dag]	42 & 58	42 & 60	42
Number of replicates	4 (WT) and 4 (OE)	4 (WT) and 4 (OE)	3 (OE), 2 (WT)
Cylinder material	PVC	PVC	PVC
Number of plants per cylinder	1	1	1
**Acquisition parameters**			
Height of scanned part of root system [cm]	18	18	15
Height of analyzed part of root system [cm]	16	18	15
Voxel size [mm]	0.07	0.07	0.05
Binning	2x2	2x2	2x2
Current [µA]	530	530	400
Voltage [kV]	130	130	120
Number of images per subscan	1600	1600	1600
Averaged images	1	1	1
Skipped images	0	0	0
Filtering [mm copper]	0.1	0.1	0.1
Observation ROI option	no	no	no
Exposure time per image (milliseconds)	200	200	200
Scan duration per subscan [s]	320	320	320
Multiscan and number of subscans	yes (3)	yes (5)	yes (5)
**Reconstruction parameters**			
Downscaling to unsigned 16 bit	yes	yes	yes
Reference ROI	no	no	no
Auto scan optimizer	yes	yes	yes
Beam hardening correction	assuming different materials, value 4	assuming different materials, value 4	assuming different materials, value 4

Methods S1 Supplementary material and methods.

Mycorrhization quantification

To visualize and quantify root colonization by AMF, roots were washed in tap water and boiled for 10 min in 10% KOH. After rinsing the roots with ddH_2O, they were boiled for 10 min in a solution containing 5% black ink and 5% acetic acid. They were washed, de-stained and stored in 5% acetic acid at 4° C. Mycorrhization was quantified by determining the mycorrhization events along the analyzed root fragments. The lid of a petri dish was carved with a grid of 4 mm^2. The roots were spread evenly on the grid. Screening was accomplished with a binocular. Quantification of mycorrhization was calculated as the proportion of positive events (only roots with clear intra-radical structures such as coiled cortical hyphae, arbuscules or vesicles) present along the line intersecting the root segment. Mycorrhization was quantified on middle root segments (approx. 4 cm^2) from three different PDR1 OE lines.

Quantitative PCR

RNA was extracted with the RNeasy Plant Mini Kit (Qiagen) and cDNA was synthesized with polydT oligonucleotides (Promega) and M-MLV reverse transcriptase (Promega) for 1 h at 55 °C. Quantitative PCR was performed in SYBR Green PCR Master Mix (Applied Biosystems) with a 7500 Fast Real-Time PCR System (Applied Biosystems). Expression levels were normalized to glyceraldehyde-3-phosphate dehydrogenase and calculated with the comparative ΔΔct method. The following primers (5' to 3') were used:

DAD1_F	*CATAGCCGTGACACCACC*
DAD1_R	*GCAACCATGCAGTCCATAGG*
MAX1_F	*GTCTAAAAACTGCACTGTTCTTG*
MAX1_R	*TTGTTTCTCTTGATGGAGAAGGAG*
PDR1_F	*CCTGAGGTTTACCAAATGGG*
PDR1_R	*GATGGTATTGGATTGGAGCA*
GAPDH_F	*GACTGGAGAGGTGGAAGAGC*
GAPDH_R	*CCGTTAAGAGCTGGGAGAAC*
ORE1-like_F	*AAAGACAGCAAAGAATGACTGG*
ORE1-like_R	*GAGCAGGGAAATTAGCAGAG*
SAG12-like_F	*TTGGTACACGACCTTACAAACTG*
SAG12-like_R	*CAACAACATCCACATTGTCCT*

SAG13-like_F	*TATGCCCGTCTCTTTAGTACC*
SAG13-like_R	*TTCGAGCAAATGTGTTACCA*
GiTUB_1F	*CCAACTTATGGCGATCTCAACA*
GiTUB_1R	*AAGACGTGGAAAAGGCACCA*

GiTUB_2 forward and reverse primers are from (Alkan et al., 2004). *PhPT3* and *PhPT5* forward and reverse primers are from (Wegmüller et al., 2008)

Gene expression was quantified in three different PDR1 OE lines. Gene expression in roots was analyzed 10 days after germination on ½ MS plates as described above to easily remove the root tips out of agarose plates. For the quantification of phosphate transporters and mycorrhizal markers, gene expression was analyzed in roots cleaned out of natural soil mix or clay 6 and 8 weeks after germination. For gene expression analysis in shoots (leaves and stems), tissue was collected from adult plants (8 weeks after germination, w.a.g.) grown on natural soil mix. This timing choice was necessary for collecting shoot tissues positive for PDR1 expression at the base of lateral dormant buds, see (Kretzschmar et al., 2012) or because of the appearance of the first senescing leaves.

^{3}H-GR24 leaf transport

Same-size leaves were collected from PDR1 OE, *pdr1* ko and relative wildtype plants grown on natural soil mix. Immediately after cutting, the petioles were submerged in 2 ml of ½ MS medium containing 0.1 μCi of ^{3}H-GR24. Leaves were incubated overnight at greenhouse conditions. The next day, each leaf petiole was washed with water and the leaf transferred in fresh 2 ml ½ MS medium and incubated for 8 hours at greenhouse conditions. Leaves and medium were then separately collected. Decays per minute (DPM) were quantified in leaves (leaf content) and medium (unloaded sap) in a Tri-Carb counter for 2 minutes after adding 3 ml of ULTIMA-Gold scintillation cocktail.

LC-MS analysis

The solvents used were LC-MS/MS grade (Chemie Brunschwig, Basel, Switzerland) and GR24 was purchased from Chiralix (Netherlands).

GR24 leaf export quantification

Green leaves from 8 week old plants were cut and incubated in 800 µL of 1 µM GR24 for 12 hours at greenhouse conditions. The next day, the leaf petioles were washed, the leftover collected (Time 0) and the leaves incubated in water for 10 additional hours at greenhouse conditions. The leaf exudates were then collected (Time 1) and all samples lyophilized. The samples were solubilized in acetonitrile and transferred to liquid chromatography (LC) vials and GR24 quantification was performed using an UPLC (Thermo Scientific Dionex UltiMate 3000) coupled to a Bruker Compact Electrospray Ionization-Quadrupole-Time-of-Flight (ESI-Q-TOF; Bruker Daltonics). The UPLC separation was performed with a C18 reverse-phase column (ACQUITY UPLC TM BEH C18, 1.7µm, 2.1 x 150mm; Waters) at 28°C using the following gradient of solvent B (acetonitrile with 0.1% [v/v] formic acid) and solvent A (water with 0.1% [v/v] formic acid): 0-0.5 min, 5% B; 0.5-12 min, 5-100% B; 12-14 min, 100% B; 14-16min, 100-5% B. The flow rate was set up to 0.3 mL min-1 and 5 µL of each sample was injected. ESI source was operated in positive mode and parameters were set as follow: gas temperature, 220°C; drying gas, 9L/min-1; nebulizer, 2.2 Bar; capillary voltage, 4500V; end plate offset, 500V. The instrument was set to acquire m/z 50-1300. Conditions for MS/MS were set as described by Christ et al. (2016). All data were analysed using Data Analysis (version 4.2, Bruker Daltonics) and TargetAnalysis (version 1.3, Bruker Daltonics). Absolute GR24 quantification was based on a standard curve between 0.05-1µg. This analysis was performed using QuantAnalysis software (version 2.2 Bruker Daltonics).

Phyllobilins extraction and analysis

Leaves from 3 month old petunia PDR1 OE and wildtype plants (this time point necessary to detect leaf senescence) were collected and immediately frozen in liquid nitrogen and grinded. One hundred mg of material was then aliquoted for phyllobilins extraction as described (Christ et al. 2016). The plant extracts were analysed by UPLC-ESI-Q-TOF as above for the GR24 leaf export experiment. We specifically looked for two phyllobilins: NCC_806 and NCC_892 (Berghold et al. 2004; Christ et al. 2016). All data were analysed using Data Analysis (version 4.2, Bruker Daltonics) and TargetAnalysis (version 1.3, Bruker Daltonics).

SL extraction and Phelipanche ramosa germination assay

SL extraction from *P. hybrida* tissues via ethyl-acetate was carried out as described (Kohlen et al., 2011). The starting material consisted of 3 to 4 grams of roots or shoots (FW) of 20 to 30 1-month-old plantlets collected from clay pots. For stems and leaves, the materials were collected from 8 week-old plants, when leaf expansion is complete and dormant buds are visible on the stem axils. The samples were pooled for each biological replicate (n=3) and technical replicate (n = 2). Seeds of *Phelipanche ramosa* were surface sterilized for 5 minutes in 2% sodium hypochlorite and 0.02% Tween-20, followed by 5 times washing with ddH_2O. The seeds were spread on two layers of filter paper (moistened with 2.4 ml ddH_2O) in a petri dish. The petri dish was sealed with Parafilm, wrapped in aluminum foil and kept at 21 °C for 12 d. The filter papers were cut into 4 pieces and transferred to separate petri dishes. 1 ml root extract (diluted $1:10^{-3}$ or $1:10^{-5}$) was added onto the filter paper. 1 µM GR24 was used as positive control for roots, 1 nM GR24 for shoots. The petri dishes were sealed with Parafilm and incubated in the dark at 25 °C. Germination rates were quantified after 5 days.

P and Pi quantifications

The pH of the different tested substrates, either plus or minus *P. hybrida* plants, was in a range of 6 to 7. Therefore Pi (inorganic phosphate) was extracted with de-ionized water and mechanical shaking (120 rpm for 1 hour) and quantified with malachite green (V.V.A.A., 1996). For this 10 ml of soil were extracted with 100 ml of water. This Pi extraction method with water included a strong and prolonged shaking of the substrates, thus this process causes soft soil fragmentation when mixed with harder particles such as bricks or clay. Hence, substrate mixes could not be directly quantified for Pi contents and were instead calculated based on the values obtained by the single components.

P quantifications in plant tissues followed published procedures (Lahner et al., 2003; Nanamori et al., 2004; Kazumi, 2016). Inductively coupled plasma mass spectrometry (ICP-MS) measurements used for P quantifications were performed with an Agilent QQQ 8800 Triple quad ICP-MS spectrometer, equipped with a standard x-lens setting, nickel cones and a “micro-mist” quartz nebulizer. All solutions were prepared from 60%

HNO_3 (Merck 1.1518.1000 ultrapure) and/or Milli-Q water. P was measured against serial dilutions of single element standards (phosphorous: Merck 1.70340.0100 in water). Indium and aluminium (100 ppb in 2% HNO_3) were used as internal standard. In a mass-shift, P was measured in “oxygen-mode” as mass (m/z 47, PO^+) resulting from the reaction of $^{31}P^+ + {}^{16}O_2$(cell gas) → $^{31}P^{16}O^+$ + O and taking place in the reaction cell. Reacting P^+ with O_2 cell gas shifts P away from the interfering ions such as $^{14}N^{16}O^{1}H^+$, $^{15}N^{16}O^+$, and $^{14}N^{17}O^+$ with $^{31}P^+$ and allowed measuring it as the PO^+ product ion at m/z 47. Values are reported as the average of 30 sweeps x 5 replicates. The RF power was 1550 W. The feed was 0.3 ml analyte/min (as aqueous 2% HNO_3)

Light sheet microscopy

Light sheet fluorescence microscopy is well suited for long-term observation of plants (Stelzer, 2015). We used the *monolithic Digital Scanned Light Sheet Microscope* (mDSLM), which is an improved version of the described DSLM by (Keller et al., 2010) and adapted the sample preparation methods that allow to capture main and lateral root growth over several days close to natural growth conditions (Maizel et al., 2011; von Wangenheim et al., 2016).

Microscopy

Sample observation (stem cuts, root and root hairs) was performed with a Nikon type 120 binocular, or a Leica DMR microscope or Zeiss microscopes using differential interference contrast optics. Images were recorded with a Leica 300F camera. Propidium iodide staining of root tips was performed as in (Sasse et al., 2015).

X-ray computed tomography and data analysis

X-ray computed tomography has proven a powerful tool for analyzing root system architectures (De Smet et al., 2012). X-ray computed tomography multiscans were performed at the Swiss Federal Institute of Technology Zurich (ETH Zürich, Switzerland) using a phoenix v|tome|x s 240 X-ray scanner (GE Sensing & Inspection Technologies GmbH, Germany). Two different configurations of acquisition parameters for tomography were chosen for the two different pot sizes (Table S1). Volumes were

reconstructed using the software datos|x (GE Sensing & Inspection Technologies GmbH, Wunstorf, Germany). For reconstruction (in 32-bit float) an auto-scan optimization and a beam hardening correction were done. Root segmentation and root system architecture analysis was performed as described previously (Pfeifer et al., 2015) using a size threshold for noise elimination of 2000 connected voxels.

Statistical methods

The sample number in this manuscript represents biological replicates. For adult *P. hybrida* plant sampling (for qPCR), tissues from 4 to 8 plants were pooled for each qPCR biological replicate (see Supplementary Information Table S2 for details). For young *P. hybrida* plant sampling (for SL extraction), tissues from 80 to 120 seedlings were pooled for each biological replicate. For young *P. hybrida* plant sampling (for root hair morphology analyses), tissues from at least 5 plants were quantified. Datasets were collected from at least 2 different times of the year, and from two different PDR1 OE lines (see Material and Methods, Plant growth section). Student's t-test is the statistical method applied to calculate if the quantified differences were or not significant (see Table S2): no * = $p > 0.05$; * = $p < 0.05$; ** = $p < 0.005$; *** = $p < 0.0005$. Significance tests were designed to compare two different genotypes (WT background and transgenic PDR1 OE or *pdr1* ko mutant) in the same growth condition. Tests are always two-sided. Values are mean +/- SE.

References

Alkan N, Gadkar V, Coburn J, Yarden O, Kapulnik Y. 2004. Quantification of the arbuscular mycorrhizal fungus *Glomus intraradices* in host tissue using real-time polymerase chain reaction. *New Phytologist* **161**(3): 877-885.

Berghold J, Eichmuller C, Hortensteiner S, Krautler B. 2004. Chlorophyll breakdown in tobacco: on the structure of two nonfluorescent chlorophyll catabolites. Chem Biodivers **1**(4): 657-668.

Christ B, Hauenstein M, Hortensteiner S. 2016. A liquid chromatography-mass spectrometry platform for the analysis of phyllobilins, the major degradation products of chlorophyll in *Arabidopsis thaliana*. *Plant J* 88(3): 505-518.

De Smet I, White PJ, Bengough AG, Dupuy L, Parizot B, Casimiro I, Heidstra R, Laskowski M, Lepetit M, Hochholdinger F, *et al.* 2012. Analyzing lateral root development: how to move forward. *Plant Cell* **24**(1): 15-20.

Kazumi N. 2016. Ultra-low level determination of phosphorus, sulfur, silicon and chlorine using the Agilent 8900 ICP-QQQ *Agilent Application note 5991-6852EN*

Keller PJ, Schmidt AD, Santella A, Khairy K, Bao Z, Wittbrodt J, Stelzer EH. 2010. Fast, high-contrast imaging of animal development with scanned light sheet-based structured-illumination microscopy. *Nat Methods* **7**(8): 637-642.

Kohlen W, Charnikhova T, Liu Q, Bours R, Domagalska MA, Beguerie S, Verstappen F, Leyser O, Bouwmeester H, Ruyter-Spira C. 2011. Strigolactones are transported through the xylem and play a key role in shoot architectural response to phosphate deficiency in nonarbuscular mycorrhizal host Arabidopsis. *Plant Physiol* **155**(2): 974-987.

Kretzschmar T, Kohlen W, Sasse J, Borghi L, Schlegel M, Bachelier JB, Reinhardt D, Bours R, Bouwmeester HJ, Martinoia E. 2012. A petunia ABC protein controls strigolactone-dependent symbiotic signalling and branching. *Nature* **483**(7389): 341-U135.

Lahner B, Gong J, Mahmoudian M, Smith EL, Abid KB, Rogers EE, Guerinot ML, Harper JF, Ward JM, McIntyre L, *et al.* 2003. Genomic scale profiling of nutrient

and trace elements in *Arabidopsis thaliana*. *Nature Biotechnology* **21**(10): 1215-1221.

Maizel A, von Wangenheim D, Federici F, Haseloff J, Stelzer EH. 2011. High-resolution live imaging of plant growth in near physiological bright conditions using light sheet fluorescence microscopy. *Plant J* **68**(2): 377-385.

Nanamori M, Shinano T, Wasaki J, Yamamura T, Rao IM, Osaki M. 2004. Low phosphorus tolerance mechanisms: phosphorus recycling and photosynthate partitioning in the tropical forage grass, Brachiaria hybrid cultivar Mulato compared with rice. *Plant Cell Physiol* **45**(4): 460-469.

Pfeifer J, Kirchgessner N, Colombi T, Walter A. 2015. Rapid phenotyping of crop root systems in undisturbed field soils using X-ray computed tomography. *Plant Methods* **11**: 41.

Sasse J, Simon S, Gubeli C, Liu GW, Cheng X, Friml J, Bouwmeester H, Martinoia E, Borghi L. 2015. Asymmetric localizations of the ABC transporter PaPDR1 trace paths of directional strigolactone transport. *Curr Biol* **25**(5): 647-655.

Stelzer EH. 2015. Light-sheet fluorescence microscopy for quantitative biology. *Nat Methods* **12**(1): 23-26.

von Wangenheim D, Fangerau J, Schmitz A, Smith RS, Leitte H, Stelzer EH, Maizel A. 2016. Rules and self-organizing properties of post-embryonic plant organ cell division patterns. *Curr Biol* **26**(4): 439-449.

V.V.A.A. 1996. *Schweizerische Referenzmethoden der Eidg. landwirtschaftlichen Forschungsanstalten*. Zürich: Zürich-Reckenholz : Eidg. Forschungsanstalt für Landwirtschaftlichen Pflanzenbau, FAP.

Wegmuller S, Svistoonoff S, Reinhardt D, Stuurman J, Amrhein N, Bucher M. 2008. A transgenic dTph1 insertional mutagenesis system for forward genetics in mycorrhizal phosphate transport of Petunia. *Plant J* 54(6): 1115-1127.
